# The CJIE1 prophage of *Campylobacter jejuni* affects protein expression in growth media with and without bile salts

**DOI:** 10.1186/1471-2180-14-70

**Published:** 2014-03-19

**Authors:** Clifford G Clark, Patrick M Chong, Stuart J McCorrister, Philippe Simon, Matthew Walker, David M Lee, Kimberly Nguy, Keding Cheng, Matthew W Gilmour, Garrett R Westmacott

**Affiliations:** 1 Enterics Research Section, Bacteriology and Enterics Program, National Microbiology Laboratory, Public Health Agency of Canada, 1015 Arlington St, Winnipeg, Manitoba R3E 3R2, Canada; 2 Mass Spectrometry and Proteomics Core Facility, National Microbiology Laboratory, Public Health Agency of Canada, 1015 Arlington St, Winnipeg, Manitoba R3E 3R2, Canada; 3 Department of Medical Microbiology, University of Manitoba, Room 543 – 745 Bannatyne Avenue, Winnipeg, Manitoba R3E 3J9, Canada; 4 Special Pathogens Program, National Microbiology Laboratory, Public Health Agency of Canada, 1015 Arlington St, Winnipeg, Manitoba R3E 3R2, Canada; 5 Current address: Faculty of Pharmacy, Apotex Centre, University of Manitoba, Winnipeg, Manitoba R3E 0T5, Canada

**Keywords:** *Campylobacter jejuni*, Prophage, Proteomics, iTRAQ, Bile response, Iron acquisition

## Abstract

**Background:**

The presence of *Campylobacter jejuni* temperate bacteriophages has increasingly been associated with specific biological effects. It has recently been demonstrated that the presence of the prophage CJIE1 is associated with increased adherence and invasion of *C. jejuni* isolates in cell culture assays.

**Results:**

Quantitative comparative proteomics experiments were undertaken using three closely related isolates with CJIE1 and one isolate without CJIE1 to determine whether there was a corresponding difference in protein expression levels. Initial experiments indicated that about 2% of the total proteins characterized were expressed at different levels in isolates with or without the prophage. Some of these proteins regulated by the presence of CJIE1 were associated with virulence or regulatory functions. Additional experiments were conducted using *C. jejuni* isolates with and without CJIE1 grown on four different media: Mueller Hinton (MH) media containing blood; MH media containing 0.1% sodium deoxycholate, which is thought to result in increased expression of virulence proteins; MH media containing 2.5% Oxgall; and MHwithout additives. These experiments provided further evidence that CJIE1 affected protein expression, including virulence-associated proteins. They also demonstrated a general bile response involving a majority of the proteome and clearly showed the induction of almost all proteins known to be involved with iron acquisition. The data have been deposited to the ProteomeXchange with identifiers PXD000798, PXD000799, PXD000800, and PXD000801.

**Conclusion:**

The presence of the CJIE1 prophage was associated with differences in protein expression levels under different conditions. Further work is required to determine what genes are involved in causing this phenomenon.

## Background

Accumulating evidence suggests that *C. jejuni* prophages CJIE1, CJIE2, and CJIE4, like the prophages of other enteric bacteria, can have a profound influence on the virulence and biology of the organism. Expression of prophage-encoded DNAses dramatically reduces the rate of natural transformation [1,2], and the presence of homologs of the CJIE1 prophage is associated with increases in adhesion and invasion [3]. In addition to the *dns* gene responsible for the production of the extracellular DNAse, proteins encoded by CJIE1 that could influence infection of hosts or survival in the environment include a putative secreted phospholipase A2 (CJE0229 homolog), a DNA adenine methylase (CJE0220 homolog), a number of hypothetical proteins with unknown functions, and two overlapping novel prophage cargo genes producing proteins provisionally designated ORF10 and ORF11 [4]. We have so far been unable to obtain infectious phage particles by induction of the CJIE1 prophage that would be useful for the creation of isogenic strains, and are not aware that any other groups have been successful either. It would be of interest to determine whether any of these prophage-encoded proteins were actually expressed and whether the presence of the prophage affected expression of other bacterial proteins.

Most protein identification experiments for *C. jejuni* have utilized one- or two-dimensional gel electrophoresis followed by mass spectroscopy of excised spots to identify expression changes specific peptides or proteins [5-10]. Recent advances in quantitative proteomic characterization include relative protein quantification by stable isotope labelling of proteins or peptides and label-free quantification [11]. Label-free quantitation of peptides/proteins using spectral counting has been used successfully for *E. coli*[12]. A method of stable isotope labelling, isobaric Tags for Relative and Absolute Quantitation (iTRAQ), has been described for quantitation of protein expression levels across multiple samples [13]. iTRAQ labelling has been used successfully for assessing proteome changes in *Acinetobacter baumanii*[14].

A previous report indicated that, for a subset of the known *C. jejuni* virulence genes, expression could be induced by growth of the organism on Mueller-Hinton agar containing 0.1% sodium deoxycholate [15]. In contrast, inclusion of 2.5% ox-bile in liquid medium significantly inhibited growth of *C. jejuni* and caused changes in protein expression [16] that suggested a specific bile stress response [17], but did not appear to cause discernable up- or down-regulation of virulence proteins.

Previous work in our laboratory indicated that the presence of CJIE1 prophage in the closely related *C. jejuni* isolates 00–2425, 00–2538, and 00–2544 was associated with higher adherence and invasion compared with the closely related isolate 00–2426, which lacked CJIE1 [3]. In the current research reported here, comparative proteomics expression experiments were undertaken in rich medium to determine whether CJIE1 proteins were differentially expressed and whether any changes in protein expression of the host bacterium could be detected. Expression of a small number of proteins, including CJIE1 proteins was detected. Additional experiments were then performed, including growth on bile salts, to determine whether the effects of CJIE1 could be detected in other growth conditions. There was evidence for CJIE1 regulation of protein expression in all growth conditions used. Furthermore, these experiments provide evidence for the existence of a general bile response and a characterization of the response to different levels of iron.

## Results

### Differential expression of proteins in isolates with and without the CJIE1 prophage in rich medium, experiment 1

Initial experiments were undertaken to detect any differences in protein expression between the four closely related isolates with (isolates 00-2425, 00-2538, and 00-2544) and without (isolate 00-2426) the CJIE1 prophage. As this experiment was undertaken before it became possible to perform whole genome sequencing of the isolates, three isolates carrying CJIE1 were compared with one isolate lacking the prophage in order to compensate for any differences among isolates and for consistency with previous work [3]. Mueller-Hinton (MH) agar + blood, a nutrient-rich medium, was used to grow *C. jejuni* for protein extraction. Scaffold analysis identified a total of 1391 total proteins at a 0.3% protein false discovery rate (FDR).

Altogether, 22 proteins were differentially expressed at a greater than 1.5-fold change (log2 change > 0.6) and *P <* 0.05 in the three isolates carrying CJIE1 compared to the single isolate without CJIE1 (Table 1; Additional file 1). Prophage structural proteins (CJE0226, CJE0227, CJE0246), two phage-associated repressors (CJE0215, signal peptidase associated with PanB), and a prophage-associated hypothetical protein (ORF7), were detected only in isolates carrying CJIE1 (Table 1), indicating that the prophage may have been induced to some extent on rich medium and providing further support indicating that these isolates differ only in the presence of CJIE1 [3,18]. The CJE0215 repressor protein was not detected in all biological replicates, suggesting that this protein may have been expressed at levels close to the threshold of detection in these experiments. Proteins encoded by two CJIE1 cargo genes, ORF 11 and the extracellular DNase (CJE0256), were expressed by the three isolates carrying CJIE1 but were not detected in isolate 00–2426. These results verified that the experimental protocols were capable of detecting differences in protein expression associated with the presence or absence of CJIE1.

**Table 1:** **Proteins regulated with ≥0.6 log**_**2 **_**change in three isolates carrying CJIE1 versus the isolate without CJIE1 when grown on MH + 10% sheep blood**

**Protein name**	**GI number**	**Locus in NCTC11168**^**a**^	**Protein function**	**log**_**2 **_**change**^**b**^
CJIE1 phage repressor protein CJE0215	gi|57237226	Not present	Maintenance of lysogeny	3.10 ± 1.27*
CJIE1 hypothetical protein CJE0246	gi|315124241	Not present	CJIE1 prophage capsid protein	2.95 ± 0.07*
CJIE1 00–2425 ORF 11	gi|313116388	Not present	Unknown	2.67 ± 0.65
CJIE1 extracellular deoxyribonuclease CJE0256	gi|57237266	Not present	Degradation of DNA	2.3 ± 0.53
CJIE1 phage major tail tube protein CJE0226	gi|315124254	Not present	Prophage structural protein	2.20 ± 0*
CJIE1 major tail sheath protein CJE0227	gi|315124253	Not present	Prophage structural protein	2.20 (single value)
CJIE1 signal peptidase I associated with PanB	gi|315124225	Not present	Prophage repressor	1.57 ± 0.78
CJIE1 hypothetical protein ICDCCJ07001_659 ORF7	gi|315124224	Not present	Unknown	2.95 ± 0.35*
Flagellar protein FlaG	gi|157414828	Cj0547	Flagellar protein	−0.77 ± 0.76
Flagellar protein FliS	gi|121612919	Cj0549	Flagellin specific chaperone	−1.00 ± 0.44
Invasion phenotype protein CipA	gi|157414972	Cj0685c	Sugar transferase	2.97 ± 0.40
Phosphate acetyltransferase Pta	gi|121613717	Cj0688	Acetate metabolism, regulation	2.30 ± 0.26
Acetate kinase AckA	gi|57236992	Cj0689	Acetate metabolism, regulation	2.00 ± 0.26
Integral membrane protein CstA	gi|218562536	Cj0917c	Carbon starvation protein	−0.73 ± 0.42
Aspartate carbamoyltransferase catalytic subunit PyrB	gi|218562712	Cj1098	Pyrimidine biosynthesis	1.66 ± 0.10
Oligoendopeptidase F PepF	gi|218562713	Cj1099	Degradation of macromolecules	1.73 ± 0.31
Hypothetical protein CJJ81176_1118	gi|121613077	Cj1100	Unknown	1.50 ± 0.44
ATP-dependent DNA helicase UvrD	gi|121612511	Cj1101	DNA helicase	1.70 ± 0.35
Hypothetical protein CJE1504	gi|57238362	Cj1305c	Unknown	−0.93 ± 0.4
Hypothetical protein ICDCCJ07001_1249	gi|315124755	Cj1305c	Unknown	−2.70 ± 0.85*
Methyltransferase protein	gi|218563030	Cj1426c	Part of capsule locus	−1.23 ± 0.38
Hypothetical protein Cj1429c	gi|218563033	Cj1429c	Capsule biosynthesis	1.73 ± 1.33

^a^GI numbers were retrieved by the Mascot software used for peptide identification; loci in NCTC 11168 were found by blastp searches by searching the NCBI non-redundant protein database using the protein sequence associated with the GI number.

^b^Three replicate experiments were done. Some experiments returned values for the protein in only 2/3 experiments; these are marked with a ‘*’. Otherwise the mean + SD log_2_ change was calculated using all three values. Experiments in which a protein expression level was identified in only one or three biological replicates are indicated with the term “single value”. Positive values indicate that proteins were upregulated in isolates carrying CJIE1 and negative values indicate proteins were downregulated in isolates carrying CJIE1 when compared with isolate 00–2426 lacking the CJIE1 prophage.

Fourteen non-prophage proteins were detected at different levels (differentially expressed) depending on the presence and absence of the prophage in the isolates tested (Table 1). These proteins included two flagellar proteins, two proteins within the capsule locus, the *Campylobacter* invasion phenotype protein, two proteins associated with acetate metabolism and regulation of the acetate switch, and other proteins having diverse functions (Table 1). Two regions of the chromosome, Cj0685-Cj0689 and CJ1098-Cj1102, appeared to be disproportionately represented. Most or all proteins encoded by contiguous genes within each of these two regions showed differential expression depending on the presence or absence of the CJIE1 prophage. Only about 2% of the proteins detected appeared to be significantly regulated by the presence of the CJIE1 prophage.

PCR using primers internal to six genes (Table S1 in Additional file 2) that were highly down-regulated in isolate 00–2426 (up-regulated in isolates carrying CJIE1) produced amplicons of the correct size, and DNA sequencing confirmed their identity. The observed regulation was therefore not due to absence of the genes. Additional PCR analysis for selected loci was performed using primers that amplified the complete coding sequence of each gene, as well as the region upstream of each gene (Additional file 2: Table S1). No differences were seen between isolate 00–2425 and 00–2426 in DNA sequences upstream of the start sites for the proteins Cj1429c (429 bp upstream sequence obtained), FliS (378 bp), UvrD (149 bp), invasion phenotype protein (391 bp), and methyltransferase protein (146 bp). These results were later confirmed by whole genome sequencing (data not shown).

Whole genome sequence data (unpublished data) demonstrated the presence of differences in homopolymeric tract lengths. One such homopolymeric tract length difference was detected in the gene encoding Cj0685 (CipA) only in isolate 00–2426 (CJIE1^-^) and resulted in truncation of the protein. For the locus encoding protein Cj1305c/CJE1505, only isolate 00–2426 contained a homopolymeric tract that would result in expression of the full-length protein, while the experimentally determined DNA sequences of the other three CJIE^+^ isolates would produce a truncated protein. The Cj1426c locus exhibited the same homopolymeric tract frameshift truncating the protein in all four isolates, while Cj1429 carried the homopolymeric tract frameshift truncating the protein in isolates 00–2425 (CJIE1^+^), 00–2544 (CJIE1^+^), and 00–2426 (CJIE1^−^), but not in isolate 00–2538 (CJIE1^+^). The loci Cj0549 and Cj1098-1191 were all capable of encoding full-length proteins in all isolates, but expressed different levels of those proteins in different isolates. Finally, the product of Cj0547, FlaG, was detected in all isolates except 00–2544. Only the 00–2544 locus had a homopolymeric tract frameshift that would truncate the protein, which appears to have resulted in a loss of expression of this protein.

### Comparison of *C. jejuni* on two additional media, MH and MH + SD, demonstrated similar patterns of protein expression, experiment 2

The protein expression patterns detected in the first experiment could have been an artifact of the rich growth medium and of little biological significance. Furthermore, guidelines for proteomics experiments have suggested that, among other things, *C. jejuni* should be grown for proteomics experiments on solid medium in the presence of sodium deoxycholate [19]. Additional experiments were therefore undertaken to explore the effect(s) of growth on different media on expression of proteins.

To enable direct comparison of CJIE1^+^ and CJIE1^−^ isolates on two different growth media in a 4-plex iTRAQ experiment, isolates 00–2425 and 00–2426 were each grown on Mueller-Hinton agar (MH) and on Mueller-Hinton agar containing 0.1% sodium deoxycholate (MH + SD). Compared to MH + blood, MH is reduced in nutrients, while growth on MH + SD has previously been associated with increased expression of specific virulence-associated proteins [15]. Proteins were obtained from lysed cells, digested, iTRAQ labelled, and subjected to 2D-LC-MS/MS analysis as described in the Materials and Methods.

Scaffold analysis indicated that 1340 proteins were detected with a 7.8% protein FDR. After removal of non-prokaryotic proteins and proteins lacking values for all fields there were 1225 proteins used for analysis.

A total of 25 proteins were differentially expressed in these experiments (Table 2; Additional file 3); all had P values of <0.05 in the Permutation test (see Additional file 3) except for hypothetical protein ICDCCJ07001_1249, for which differences were not statistically significant at 75% (*P* = 0.20). Of the eight CJIE1 prophage-associated proteins differentially expressed during growth on MH + blood (previous experiment) only two were differentially expressed during growth on MH and MH + SD, suggesting there may be less induction of prophage proteins on more nutrient-limited medium. Of the non-prophage proteins differentially expressed during growth on MH + blood (Table 1), nine were differentially expressed during growth on MH agar and MH + SD agar (see protein names in bold, Table 2). These proteins were in the regions of contiguous genes (Cj0685-Cj0689 and CJ1098-Cj1102) and within the capsule locus, as noted in the first set of experiments (Table 1). An additional protein encoded by a gene within the capsule locus, the sugar transferase Cj1421, was also differentially expressed in this set of experiments; there was a suggestion that this may also have been the case in the earlier experiment using MH + blood, but high variability between replicates precluded drawing any definitive conclusions (data not shown). The other 14 proteins that were clearly differentially expressed when grown on MH +/− SD did not exhibit equivalent differential expression when grown on MH + blood (see Table 2). Similarly, 10 proteins exhibiting differential expression on MH + blood did not appear to have detectable differences in expression between isolates 00–2425 and 00–2426 when grown on MH or MH + SD.

**Table 2:** **Proteins regulated with ≥0.6 log**_**2 **_**change in isolate 00–2426 (no CJIE1) compared with isolate 00–2425 (with CJIE1, grown on MH) as reference when isolates were grown on MH and MH + SD**

**Protein name**^**a**^	**GI number**	**Locus in NCTC 11168**	**Protein function or region**	**log**_**2 **_**change 00–2425 MH + SD agar**	**log**_**2 **_**change 00–2426 MH agar**	**log**_**2 **_**change 00–2426 MH + SD agar**
**CJIE1 00–2425 ORF 11**	gi|313116388	Not present	Unknown	0.67 ± 0.40	−2.43 ± 0.70	−2.13 ± 1.27
**CJIE1 signal peptidase I associated with PanB**	gi|315124225	Not present	Prophage repressor	0.50 ± 0.20	−3.75 ± 0.64	−3.15 ± 0.78
Hypothetical protein CJJ81176_0110	gi|121613178	Cj0073c	Unknown	0.83 ± 0.21	0.87 ± 0.99	1.27 ± 0.67
Iron-sulfur cluster binding protein	gi|121612415	Cj0074c	Unknown	0.57 ± 0.6	0.63 ± 1.01	1.00 ± 0.61
Anaerobic C4-dicarboxylate transporter	gi|218561769	Cj0088		1.03 ± 1.04	1.13 ± 0.40	2.17 ± 0.38
Hypothetical protein Cj0170	gi|218561850	Cj0170	Unknown	−0.30 ± 0.10	−2.80 ± 0.90	−2.97 ± 0.31
Hypothetical protein CJJ81176_0447	gi|121613189	Cj0427	Unknown	0.33 ± 1.46	1.33 ± 1.12	1.07 ± 1.66
**Invasion phenotype protein CipA**	gi|157414972	Cj0685c	Sugar transferase	0.70 ± 0.20	−2.87 ± 0.21	−2.43 ± 0.47
**Phosphate acetyltransferase Pta**	gi|121613717	Cj0688	Acetate metabolism, regulation	−0.03 ± 0.21	−2.43 ± 0.31	−2.67 ± 0.58
**Acetate kinase AckA**	gi|384443065	Cj0689	Acetate metabolism, regulation	−0.33 ± 0.64	−3.37 ± 0.25	−2.70 ± 1.00
Oxidoreductase	gi|218562461	Cj0833c	Oxidoreductase	−0.10 ± 0.10	−0.96 ± 1.76	−1.10 ± 0.95
Ankyrin repeat-containing periplasmic protein	gi|218562462	Cj0834c	Mediates protein-protein interactions	−0.67 ± 0.25	−1.20 ± 2.42	−1.60 ± 1.05
Amino acid ABC transporter, ATP-binding protein PEB1	gi|121612723	Cj0922c	Amino-acid ABC transporter	0.77 ± 0.21	0.63 ± 0.68	1.03 ± 0.45
**Aspartate carbamoyltransferase catalytic subunit PyrB**	gi|218562712	Cj1098	Pyrimidine biosynthesis	0.20 ± 0.35	−1.70 ± 0.17	−1.57 ± 0.21
**Oligoendopeptidase F PepF**	gi|218562713	Cj1099	Degradation of macromolecules	−0.20 ± 0.10	−1.43 ± 0.32	−1.63 ± 0.23
**ATP-dependent DNA helicase UvrD**	gi|384448349	Cj1101	DNA helicase	0.73 ± 0.42	−1.65 ± 0.21*	−1.23 ± 0.47
**Hypothetical protein ICDCCJ07001_1249**	gi|315124755	Cj1305	Part of O-linked glycosylation locus	−0.35 ± 0.35*	1.30 ± 1.56*	2.53 ± 2.60
Hypothetical protein Cj1310c	gi|218562921	Cj1310c	Part of O-linked glycosylation locus	−0.23 ± 0.25	1.33 ± 1.02	0.87 ± 0.81
Motility accessory factor	gi|407942710	Cj1318	Part of O-linked glycosylation locus	0.03 ± 0.32	0.93 ± 0.98	1.00 ± 0.17
Maf7; hypothetical protein C8J_1258	gi|157415578	Cj1342c	Motility accessory factor	0.20 ± 0.10	1.57 ± 0.31	1.87 ± 0.31
Sugar transferase	gi|218563025	Cj1421c	Part of capsule locus	0.50 ± 0.17	0.80 ± 0.66	1.3 ± 0.60
**Methyltransferase protein**	gi|218563030	Cj1426c	Part of capsule locus	0.57 ± 0.72	1.13 ± 0.72	1.03 ± 0.65
**Hypothetical protein Cj1429c**	gi|218563033	Cj1429c	Capsule biosynthesis	0.33 ± 0.32	−2.50 ± 0.36	−1.80 ± 1.25
Aminotransferase	gi|218563040	Cj1436c	Part of capsule locus	−0.30 ± 0.30	−1.53 ± 1.26	−2.03 ± 0.25
Hypothetical protein CJJ81176_1657	gi|121612948	Cj1666c	Probable periplasmic protein	−0.63 ± 0.46	−0.63 ± 0.51	−1.43 ± 0.38

^a^Protein names in bold were also detected in previous experiments after growth on MH + 10% sheep blood. Some experiments returned values for the protein in only 2/3 experiments; these are marked with a `*'. Other information was the same as described in the footnote to Table 1.

In addition to the proteins described in the previous section, two of the proteins described in this experiment contained homopolymeric sequences. The homopolymeric tract within Cj1421 was the same length in all four isolates tested, and would result in expression of a full-length protein. The homopolymeric tract in locus Cj0170 would allow expression of full-length protein in isolates 00–2425 and 00–2538 but would cause truncation of the protein in isolates 00–2426 and 00–2544. Much more Cj0170 protein was detected in isolates 00–2425 and 00–2538 than in the latter two proteins; it seemed likely that the expression of Cj0170 correlated with the length of the homopolymeric tract and not directly with the presence of the CJIE1 prophage.

A few of the proteins in Table 2 appeared to be expressed differently when isolates were grown on MH + SD as compared to MH. For isolate 00–2425, these proteins include homologs of the CJIE1 ORF11 protein, Cj0073c, Cj0088 (though the data associated with this protein had a high standard deviation about the mean), Cj0685c, Cj0922c, and Cj1101. Similarly, growth of isolate 00–2426 on MH + SD versus MH resulted in comparatively greater amounts detected, therefore apparently higher expression, of Cj0088, Cj0689, Cj1305, and Cj1429.

### Growth of *C. jejuni* on different media revealed different patterns of expression, experiments 3 (using isolate 00–2425) and 4 (using isolate 00–2426)

A large number of proteins appeared to be differentially regulated depending on whether the culture medium contained 0.1% sodium deoxycholate or not, rather than on the presence or absence of prophage CJIE1 (see Additional file 3). A different experimental design was therefore used in experiments to further assess these observations and to confirm that proteins that appeared to be expressed at much lower levels in isolate 00–2426 were actually expressed.

We compared protein expression for each isolate (each experiment) after growth on MH + blood, MH, and MH + SD. A second bile salt preparation (2.5% Oxgall) was also included to determine which proteins were involved in a general response to bile. As previously noted [16], incorporation of 2.5% ox-bile in the MH + OX media greatly inhibited the growth of these isolates so that a greater number of culture plates were required to obtain sufficient bacterial cells for protein preparation. The expression levels of proteins from cultures grown on MH agar were used as the reference against which log_2_ expression values were calculated. The experimental design used required two separate 4-plex iTRAQ experiments, one with four different media for each isolate used, and was therefore incapable of allowing direct comparison of results from isolate 00–2425 (CJIE1^+^) to those of 00–2426 (CJIE1^−^) though it was possible to compare changes in expression levels compared with the reference.

**Figure 1:**
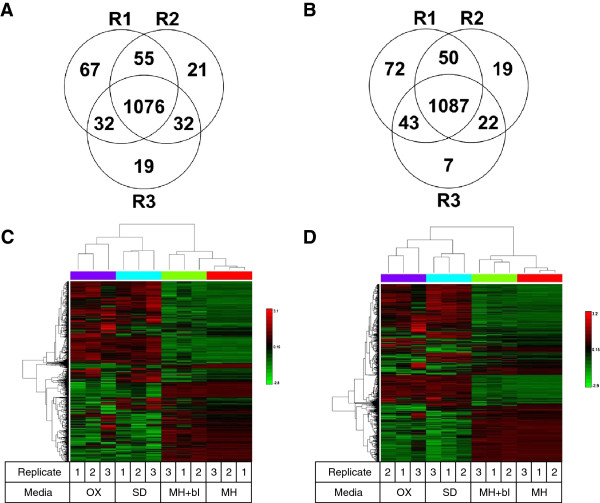
**Heat map of proteomic data in experiments comparing growth of isolates on MH + blood (MH + bl), MH, MH + SD, and MH + OX. A**. *C. jejuni* 00–2425, showing consistent expression of 1076/1302 proteins (area of overlap) identified in three biological replicate experiments. **B**. *C. jejuni* 00–2426 strain, showing consistent expression of 1087/1300 proteins (area of overlap) identified in three biological replicate experiments. Cluster analysis for proteins expressed in isolates 00–2425 **(C)** and 00–2426 **(D)**.

When data from the separate experiments were merged using the Scaffold program, there were 1464 proteins identified with a 5.0% protein FDR. Results were quite reproducible across biological replicate experiments; 83% of proteins were detected in all three replicates (Figure 1A and B). Relative protein expression levels were similar across replicates, and the majority of proteins varied consistently across replicates grown in same media (Figure 1C and D). A well-defined effect on protein induction was observed between the MH + OX and MH + SD media when compared with the MH and MH media. Both strains also exhibited clear, consistent differences in protein expression when grown in the MH + OX and MH + SD media compared to the MH + blood and MH media, though the patterns of protein expression were somewhat less reproducible in the presence of these bile salts (see Figure).

A number of proteins were expressed at detectable levels in either *C. jejuni* 00–2425 or in 00–2426, but not both, when results from these four media were compared (Table 3). Whole genome sequence data (not shown) indicated that, other than the CJIE1 loci, the genes coding for these proteins were present in both isolates and, with one exception, did not have SNPs that would completely abrogate expression. The exception was the serine/threonine transporter protein SstT, which had a SNP only in isolate 00–2426 that would result in the expression of a truncated protein (unpublished data). SstT protein expression was detected in both isolates 00–2425 and 00–2426 (Additional file 4).

**Table 3:** **Proteins detected only in *C. jejuni *** **00–2425 (carrying CJIE1) or in *C. jejuni *** **00–2426 (without CJIE1)**

**Protein designation**		**Protein name**	**GI Number**
**Detected only in isolate 00–2425 (carrying CJIE1)**			
CJIE1	Prophage indel	00-2425 hotspot protein ORF11	gi|313116388
CJIE1	Prophage	Signal peptidase I prophage repressor	gi|315124225
CJIE1	Prophage ORF7	Hypothetical protein ICDCCJ07001_659	gi|315124224
CJE0269	Prophage	Bacteriophage DNA transposition protein B, putative	gi|57237279
CJE0256	Prophage	Extracellular deoxyribonuclease	gi|57237266
CJE0246	Prophage	Hypothetical protein ICDCCJ07001_677	gi|315124241
CJE0228	Prophage	Hypothetical protein ICDCCJ07001_690	gi|315124252
CJE0226	Prophage	Phage major tail tube protein	gi|315124254
CJE0215	Prophage	Phage repressor protein	gi|157414969
Cj1620c	MutY	A/G-specific adenine glycosylase	gi|218563209
Cj1588c		putative MFS transport protein	gi|218563177
Thauera spp.		pyruvate phosphate dikinase PEP/pyruvate-binding	gi|217970436
**Detected only in isolate 00–2426 without CJIE1**			
Cj0065c	FolK	2-amino-4-hydroxy-6-hydroxymethyldihydropteridine pyrophosphokinase	gi|157414379
Cj1731c		Holliday junction resolvase	gi|121612651
Cj0566		Hypothetical protein Cj0566	gi|218562217
Cj0620		Hypothetical protein Cj0620	gi|218562271
CJE1531		Hypothetical protein CJE1531	gi|57238385
Cj1113		Hypothetical protein CJJ81176_1131	gi|121613282
Cj1193c		Hypothetical protein CJJ81176_1208	gi|121613678
CJE0735	IlvC	Ketol-acid reductoisomerase	gi|152990905

Of the 12 proteins detected only in isolate 00–2425, 9 were CJIE1 prophage proteins, an observation consistent with the presence of the prophage only in this isolate. The other three proteins expressed only in isolate 00–2425 were associated with core functions of the bacterium, and PCR analysis indicated that a product could be amplified using primers specific for the gene encoding Cj1620c in both 00–2425 and 00–2426. Proteins uniquely expressed at detectable levels in *C. jejuni* 00–2426 included 5 hypothetical proteins of unknown function. As before, PCR amplification products were obtained from both 00–2425 and 00–2426 for all non-CJIE1 loci except CJE0735, which was not tested. Together these data suggest that the differential expression of most or all of these proteins was truly due to regulation associated with the presence or absence of the CJIE1 prophage rather than loss of, or interruption within, the genes involved.

Profound differences in protein expression associated with each different media type were noted. A summary of the results from these experiments are shown in Table 4. Expression of 6 – 8% of proteins differed between isolates grown on MH + blood compared with isolates grown on MH medium (Table 4). Much higher proportions of the total proteins exhibited differential regulation when comparing MH with MH + SD (~72 – 79%) and MH with MH + OX (~69 – 70%). Between 5 – 11% of proteins appeared to be differentially expressed when MH + SD and MH + OX were compared. These results are consistent with a fairly specific, targeted response when comparing medium with and without blood; a general stress response to bile salts; and a small number of regulatory effects specific for either 0.1% sodium deoxycholate or 2.5% Oxgall. There also appeared to be a limited number of differences in protein expression observed due to the presence of CJIE1 in isolate 00–2425, detected as differences in the number of proteins differentially expressed when comparing MH and MH + SD, and MH + SD and SD + OX.

**Table 4:** **Protein expression showing ≥ log**_**2 **_**0.6 change in isolates grown on different media**

**Comparison**	**Number of proteins up-regulated**				**Total regulated**	**Total proteins**	**% of total regulated**
	**In MH**	**In MH**	**In SD**	**In OX**			
00- 2425 MH + blood vs MH	44	70			114	1366	8.3
00- 2426 MH + blood vs MH	37	53			90	1363	6.6
00-2425 MH vs SD		426	568		994	1365	72.8
00-2426 MH vs SD		463	615		1078	1362	79.1
00-2425 MH vs OX		426		531	957	1366	70.1
00-2426 MH vs OX		439		503	942	1362	69.2
00-2425 SD vs OX			124	29	153	1375	11.1
00-2426 SD vs OX			40	33	73	1375	5.3

### Proteins showed differential expression in isolates with and without CJIE1 when grown on MH + SD and MH + OX compared with MH

Differential expression was detected for several proteins when isolates were grown on MH and MH + SD media (Additional file 4, an Excel file containing a complete data set describing the third and fourth experiments). Among the proteins more highly up-regulated on MH + SD were CmeA, CmeB, CmeC, other outer membrane efflux system proteins, the iron-uptake ABC transporter ATP-binding protein CfbpC, a subset of 30S ribosomal proteins, the TlyA hemolysin, the thermonuclease family protein, a number of proteins associated with motility, PEB1 (in MH + SD only), and some of the proteins associated with cell wall/membrane/envelope biogenesis. There were also proteins whose expresson was down-regulated during growth on MH + SD relative to MH, including CiaB, PEB4, catalase, and the AhpC/Tsa family antioxidant protein. Additional proteins exhibiting differential expression in the presence of bile salts can be found in Additional file 4. A few of these proteins appeared to have increased or decreased expression from the reference condition depending on the presence or absence of the CJIE1 prophage.

### Bile salts affect the expression of proteins regulated by CJIE1

Proteins affected by the presence or absence of CJIE1, described above, were also regulated by bile salts. The invasion phenotype protein (CipA) and UvrD were both more highly expressed in both 00–2425 and 00–2426 in the presence of both 0.1% sodium deoxycholate and 2.5% Oxgall (Table 5). Hypothetical protein Cj1429 was more highly expressed at log_2_ values ≥0.6 in isolate 00–2426 in the presence of both bile salt preparations, but in isolate 00–2425 only in the presence of 2.5% Oxgall. Similarly, the aspartate carbamoyltransferase catalytic subunit was expressed at log_2_ values ≥0.6 in isolate 00–2426 in the presence of both bile salt preparations but in isolate 00–2425 only in the presence of 0.1% sodium deoxycholate. Acetate kinase and oligoendopeptidase F exhibited decreased expression at log_2_ values ≥0.6 in both isolates but only in the presence of 0.1% deoxycholate.

**Table 5:** Effect of media differing in iron concentration and inclusion of bile salts on expression of consensus proteins differentially regulated by the presence or absence of CJIE1 in initial experiments

				**Log**_**2 **_**change**			
**Protein**	**COG**	**GI number**	**Locus in NCTC 11168**	**0.1% sodium deoxycholate**		**2.5% Oxgall**	
				**00-2425**	**00-2426**	**00-2425**	**00-2426**
Invasion phenotype protein CipA	R	gi|157414972	Cj0685c	1.17 ± 0.30	1.57 ± 0.35	1.13 ± 0.46	1.20 ± 0.66
phosphate acetyltransferase Pta	R	gi|121613717	Cj0688	0.07 ± 0.30	1.10 (n = 1)	−0.93 ± 0.21	1.90 (n = 1)
Acetate kinase AckA	C	gi|57236992	Cj0689	−1.27 ± 0.10	−1.00 ± 0.60	−0.57 ± 0.50	−0.50 ± 0.36
Aspartate carbamoyltransferase catalytic subunit PyrB	F	gi|218562712	Cj1098	0.80 ± 0.30	1.00 ± 0.20	0.50 ± 0.40	0.73 ± 0.25
Oligoendopeptidase F PepF	E	gi|218562713	Cj1099	−0.87 ± 0.20	−0.90 ± 0.17	−0.03 ± 0.06	−0.13 ± 0.23
ATP-dependent DNA helicase UvrD	L	gi|121612511	Cj1101	1.20 ± 0.30	1.40 ± 0.17	1.40 ± 0.36	1.40 ± 0.10
Hypothetical protein Cj1429c	S	gi|218563033	Cj1429c	0.33 ± 0.40	1.45 ± 0.49	1.13 ± 0.25	2.5 ± 0
Methyltransferase protein		gi|218563030	Cj1426c	0.43 ± 0.30	0.97 ± 0.21	0.23 ± 0.40	0.33 ± 0.70

Expression of proteins on MH + blood was set as the reference separately for isolates 00–2425 and 00–2426 against which fold change on each of the different growth media was calculated for each isolate.

*COGs*, [C] Energy production and conversion; [E] Amino acid transport and metabolism; [F] Nucleotide transport and metabolism; [K] Transcription; [L] Replication, recombination, and repair; [Q] Secondary metabolites biosynthesis, transport and catabolism; [R] General function prediction only; [S] Function unknown.

### Bile salts induce prophages and affect expression of proteins encoded by various CJIE elements

CJIE1 affected expression of some prophage structural proteins and some cargo genes as well. As shown in Additional file 2: Table S2, a slight increase in CJIE1 prophage protein expression occurred when isolate 00–2425 was grown on MH + SD, while growth on MH + OX generally resulted in greater changes in CJIE1 structural protein expression (Additional file 2: Table S2). These changes occurred in the absence of significant changes in expression of the CJE0215 repressor protein, and despite the increased expression of the second CJIE1 repressor (signal peptidase I) located near the gene encoding PanG in the 00–2425 genome [4]. Expression of ORF11, the unique protein product of a gene carried within CJIE1, was also increased in the presence of both bile salts. In contrast, inclusion of bile salts in the growth medium resulted in a 2 – 3 fold decrease in expression of the extracellular deoxyribonuclease carried by CJIE1.

There was evidence that the expression of proteins encoded by CJIE4 was also affected by bile salts (Additional file 2: Table S2). In this case, growth of both 00–2425 and 00–2426 on medium containing bile salts resulted in a ~3 – 5 fold reduction in expression of one of the prophage repressor proteins (CJE1429), with concomitant increases in the expression of various prophage structural or functional proteins. In contrast, expression of the second prophage repressor carried by CJIE4 (CJIE1440) did not change much under different growth conditions. Increases in the expression of CJIE4 prophage structural proteins (integrase recombinase, HK97 family major capsid protein) suggested concomitant induction of the prophage in the presence of bile salts. Proteins encoded by two genes in an indel that differed among different strains (CJE1440 and CJE1441, manuscript in preparation) had decreased expression in the presence of bile salts. RloG (CJE1430) was slightly down-regulated (log^2^ change = −0.67) only in the presence of 0.1% sodium deoxycholate.

Proteins carried by two other integrated elements were expressed in these experiments (Additional file 2: Table S2). A CJIE3 cytochrome C family protein was down-regulated in the presence of bile salts, slightly less so when CJIE1 was present. Three CJIE3 proteins were annotated as having an association with branched chain amino acid transport. Expression of the ATP-binding protein did not change a great deal in the presence of bile salts, while expression of the putative permease protein was increased somewhat (Additional file 2: Table S2). The presence of CJIE1 appeared to have little effect on expression of these proteins. In contrast, the periplasmic amino acid-binding protein was strongly down-regulated in the presence of both bile salts. Only one CJIE2 hypothetical protein appeared to be expressed (Additional file 2: Table S2), and levels of this protein were increased by the presence of bile salts.

These data indicate that CJIE prophages may have been induced upon growth in bile salts. Many prophage cargo genes were expressed; differential carriage of these cargo genes could lead to differences in the biology of the host bacterium.

### Bile salts affected expression of selected virulence-associated proteins

The genes and proteins associated with virulence and pathogenesis of *C. jejuni* have been reviewed recently [20]; a subset of possible virulence genes was selected from the total for further analysis. Comparative protein expression data were reviewed to determine the effects of the CJIE1 prophage and different growth media on expression of a subset of these proteins (Additional file 2: Table S3; Additional file 4).

Proteins involved in capsule polysaccharide synthesis and transport (COG class M) were all more highly expressed when isolates were grown on medium containing bile salts. The one exception was the capsular polysaccharide transport protein, which only showed increased expression in the presence of 0.1% sodium deoxycholate and not in 2.5% Oxgall. Sialic acid synthase expression was decreased in the presence of bile salts.

Growth on medium containing bile salts resulted in increased expression of flagellin (both sources identified), the flagellar basal rod modification protein, the flagellar biosynthesis regulator FlhA (greater change in expression compared to reference in 0.1% deoxycholate compared with 2.5% Oxgall), and the flagellar biosynthesis regulator FlhF (Additional file 2: Table S3). Many other flagellar structural proteins were also expressed at higher levels in the presence of bile salts (Additional file 4). Bile salts decreased the expression of FlaC, the flagellar biosynthesis sigma factor, the flagellar basal rod modification protein, the flagellin modification protein A, and the CheY chemotaxis protein (Additional file 2: Table S3) as well as flagellar assembly proteins, two basal body proteins, the flagellar capping protein, and 2/3 flagellar motor switch proteins (Additional file 4). There was a large difference in expression of the flagellar basal rod modification protein compared with the reference state in the presence and absence of the CJIE1 prophage. Expression of the flagellin modification protein PseA was also decreased, but to a greater degree in the presence of 0.1% deoxycholate in the culture medium than of 2.5% Oxgall (Additional file 2: Table S3).

Effector proteins associated with *C. jejuni* pathogenesis also showed changes in expression in the presence of bile salts. Expression of both CiaB and PEB1 was much lower in the presence of bile salts (Additional file 2: Table S3), as was the expression of FlaC and all subunits of cytolethal distending toxins, especially subunits B and C. However, expression of the TlyA hemolysin and a flippase involved in N-linked glycosylation was increased in the presence of bile salts.

Expression of a number of regulatory proteins was also affected by growth conditions. The FliA flagellar biosynthesis sigma factor, the DnaK suppressor protein, and the GroES co-chaperonin were all expressed at much lower levels in the presence of bile salts than in reference conditions (growth on MH), while the Sigma 54-associated transcriptional regulator and DnaJ were expressed at higher levels. GroEL expression was reduced in the presence of 0.1% deoxycholate, but elevated somewhat when grown on medium containing 2.5% Oxgall; in both cases, these effects were less apparent in isolate 00–2425 carrying CJIE1.

While the levels of all virulence-associated proteins were affected to a greater or lesser degree by the presence of bile salts, only a few – most strikingly CiaB, DnaK, PEB1A, and PseA (when grown on MH + SD), and the flagellar basal rod modification protein (when grown on both media containing bile salts) – also appeared to be regulated by the presence of CJIE1.

### Growth of bacteria on medium without blood induces proteins involved in iron acquisition and oxidative stress responses

As seen in Table 4, a minority of proteins were differentially expressed during growth on MH + blood and MH. Details of these expression differences are shown in Table 6 for 99 proteins. Of the proteins regulated by iron levels (blood) in the medium, 24 were associated with iron acquisition. Proteins associated with oxidative stress defence pathways involving catalase production, the antioxidant AhpC/Tsa family protein, thioredoxin-like protein, and thioredoxin reductase, which are known to be associated with the iron acquisition pathways, were also detected (Table 6). Of the proteins that were differentially expressed in the presence or absence of blood, 8 proteins were associated with chemotaxis, molybdate transport, and methionine biosynthesis, 11 were hypothetical proteins, 3 were transporters, and 46 proteins were associated with varied or unknown functions. Expression of Per, the regulator of the oxidative stress response, was not affected by the presence or absence of blood in the medium (Additional file 4).

**Table 6:** ***C. jejuni *** **proteins responding to decreased iron concentration assessed by comparing protein expression after growth on MH + blood and on MH medium**

**Protein**	**Locus in NCTC11168**	**GI number**	**Log**_**2 **_**change in MH medium**	
			**00-2425**	**00-2426**
**Iron acquisition**				
Iron-uptake ABC transporter ATP-binding protein CfbpC, putative	Cj0173c	gi|218561852	1.27 ± 0.75	1.10 ± 0.53
Iron ABC transporter, permease CfbpB, putative	Cj0174c	gi|121612175	0.80 ± 0.71	0.90 ± 0
Iron-uptake ABC transporter, periplasmic iron-binding protein CfbpA, putative	Cj0175c	gi|218561854	2.00 ± 1.30	1.97 ± 1.27
Iron transport protein, putative	Cj0177	gi|218561856	3.30 ± 0.17	2.73 ± 1.05
TonB-denpendent outer membrane receptor, putative	Cj0178	gi|218561857	2.70 ± 0.56	2.97 ± 0.29
Biopolymer transport ExbB protein, TonB transport system	Cj0179	gi|218561858	3.03 ± 0.32	3.37 ± 1.61
Periplasmic TonB transport protein	Cj0753c	gi|315124276	3.5 (single value)	3.5 (single value)
Ferric receptor CfrA	Cj0755	gi|57237601	2.37 ± 0.15	2.23 ± 0.81
Enterochelin uptake ATP-binding protein CeuD	Cj1354	gi|218562963	2.00 ± 1.06	2.00 ± 0.56
Enterochelin ABC transporter, periplasmic enterochelin-binding protein CeuE	Cj1355	gi|121612138	1.80 ± 0.66	1.40 ± 0.26
Periplasmic protein P19	Cj1659	gi|157415879	1.97 ± 1.50	2.10 ± 1.04
Iron permease, putative	Cj1658	gi|218563246	1.97 ± 1.04	1.70 ± 1.01
Heme iron utilization protein, putative	Cj1613c	gi|121612296	3.60 ± 0.95	3.70 ± 0.5
TonB-dependent heme receptor ChuA	Cj1614	gi|57238627	3.35 ± 0.35	4.20 ± 0
TonB-dependent heme receptor ChuA	Cj1614	gi|157415833	1.80 ± 0.70	1.73 ± 0.87
TonB-dependent heme receptor ChuA	Cj1614	gi|121613006	1.20 ± 0.56	0.33 ± 0.64
Hemin ABC transporter, permease protein, putative ChuB	Cj1615	gi|153951720	3.17 ± 0.55	2.93 ± 1.19
Hemin uptake system ATP-binding protein ChuC , putative	Cj1616	gi|218563205	2.97 ± 0.74	3.07 ± 0.50
Hemin uptake system periplasmic hemin-binding protein, putative	Cj1617	gi|218563206	2.83 ± 0.59	2.53 ± 0.42
Biopolymer transport ExbD protein, TonB transport system	Cj1629	gi|153952038	1.63 ± 0.81	1.57 ± 0.49
Integral membrane protein, putative	Cj1660	gi|218563248	1.27 ± 0.81	1.5 ± 0.79
ABC transporter permease, putative	Cj1661	gi|218563249	1.67 ± 0.97	1.57 ± 0.67
Integral membrane protein, putative	Cj1662	gi|218563250	1.93 ± 1.15	1.60 ± 0.87
ABC transporter ATP-binding protein	Cj1663	gi|157415883	1.53 ± 0.80	1.53 ± 0.51
**Oxidative stress**				
Thioredoxin reductase	Cj0146c	gi|218561827	0.93 ± 0.47	0.87 ± 0.5
Antioxidant, AhpC/Tsa family	Cj0334	gi|157414631	0.97 ± 0.32	0.70 ± 0.20
Catalase	Cj1385	gi|121612952	3.37 ± 0.23	2.80 ± 0.56
Thioredoxin-like protein	Cj1664	gi|121613144	1.23 ± 0.91	1.27 ± 1.08
**Anthranilate synthesis**				
Anthranilate synthase component I	Cj0345	gi|121612335	0.80 ± 0.75	0.87 ± 0.71
Anthranilate synthase component II	Cj0346	gi|121612640	0.67 ± 0.60	0.60 ± 0.46
N-(5′phosphoribosyl)anthranilate isomerase	Cj0347	gi|157414644	1.00 ± 0.53	0.83 ± 0.65
**Chemotaxis**				
Methyl-accepting chemotaxis protein	Cj1189c	gi|121613242	0.70 ± 1.10	0.07 ± 0.06
Methyl-accepting chemotaxis protein	Cj1506c	gi|121613017	2.0 ± 1.31	3.23 ± 2.40
**Methionine biosynthesis**				
5-methyltetrahydropteroyltriglutamate--homocysteine methyltransferase MetE	Cj1201	gi|157415465	0.50 ± 0.35	1.20 ± 0.26
Homoserine O-succinyltransferase MetA	Cj1726c	gi|121613233	0.80 ± 0.35	1.53 ± 0.55
Homoserine O-acetyltransferase MetB	Cj1727c	gi|315125108	1.27 ± 0.57	1.93 ± 0.9
**Molybdate transport**				
Molybdate transport system ATP-binding protein	Cj0300c	gi|157414597	0.67 ± 0.15	0.77 ± 0.25
Hypothetical protein C8J_0279 molybdenum-pterin-binding protein	Cj0302	gi|157414599	0.30 (single value)	0.67 ± 0.21
Molybdate transport system substrate-binding protein	Cj0303	gi|157414600	0.83 ± 0.75	0.77 ± 0.25
**Hypothetical proteins**				
Hypothetical protein C8J_0062	Cj0069	gi|157414383	−0.63 ± 0.06	−0.60 ± 0.17
Hypothetical protein CJJ81176_0110	Cj0073c	gi|121613178	−0.93 ± 0.68	−0.57 ± 0.35
Hypothetical protein C8J_0189	Cj0200c	gi|157414509	0.67 ± 0.81	0.80 ± 0.78
Hypothetical protein Cj0427	Cj0427	gi|218562085	−1.47 ± 0.67	0.07 ± 0.58
Hypothetical protein CJJ81176_0676	Cj0648	gi|121613289	0.70 ± 0.28	0.50 ± 0.36
Hypothetical protein C8J_1185	Cj1242	gi|157415505	−0.60 (single value)	−1.10 ± 0.71
Hypothetical protein C8J_1529	Cj1627c	gi|157415847	0.60 ± 0.20	0.50 ± 0.61
Hypothetical protein CJJ81176_1628	Cj1637c	gi|121613390	−0.33 ± 0.15	−0.73 ± 0.35
Hypothetical protein C8J_1561	Cj1659	gi|157415879	1.97 ± 1.50	2.10 ± 1.04
Hypothetical protein CJE0598 CJIE2 prophage	not present	gi|57238304	1.10 ± 0.28	0.77 ± 0.40
Hypothetical protein CXIVA_22050	not present	gi|339443269	0.67 ± 0.57	0.50 ± 0.53
**Transporters**				
Sodium/dicarboxylate symporter	Cj0025c	gi|121612756	0.80 ± 0.66	0.40 ± 0.44
Anaerobic C4-dicarboxylate transporter	Cj0088	gi|218561769	−1.10 ± 0.61	−0.63 ± 0.23
OPT family oligopeptide transporter	Cj0204	gi|121613125	0.73 ± 0.21	0.50 ± 0.17
**Other function or unknown function**				
Non-haem iron protein	Cj0012c	gi|218561705	−0.83 ± 0.61	−0.77 ± 0.64
Cytochrome c family protein	Cj0037c	gi|121612292	0.93 ± 0.21	1.03 ± 0.35
Aspartate ammonia-lyase	Cj0087	gi|157414400	−1.10 ± 0.50	−1.1 ± 0.40
Periplasmic protein, putative	Cj0093	gi|218561774	0.80 ± 0.53	0.80 ± 0.56
Superoxide dismutase, Fe	Cj0169	gi|157414485	−1.23 ± 0.84	−1.20 ± 1.47
Superoxide dismutase, Fe	Cj0169	gi|153951398	−3.70 ± 2.95	−0.93 ± 0.97
Integral membrane protein	Cj0236c	gi|153952171	0.53 ± 0.75	0.97 ± 0.76
Chemotaxis histidine kinase	Cj0284c	gi|218561946	−0.83 ± 0.61	−0.53 ± 0.42
Ferredoxin 4Fe-4S	Cj0333c	gi|121612302	−1.10 ± 0.89	0.53 ± 0.15
Tryptophan synthase subunit alpha	Cj0349	gi|121613100	0.67 ± 0.47	0.80 ± 0.56
Cytochrome c551 peroxidase	Cj0358	gi|121612870	−0.67 ± 0.55	0.50 ± 0.17
Putative integral membrane protein	Cj0421c	gi|218562079	1.00 ± 0.71	0.73 ± 0.12
Putative periplasmic protein	Cj0425	gi|218562083	1.70 (single value)	1.10 (single value)
Succinate dehydrogenase flavoprotein subunit	Cj0437	gi|218562095	−0.70 ± 0.44	−0.6 ± 0.87
DNA polymerase III subunit epsilon	Cj0452	gi|218562107	−0.23 ± 0.21	−0.67 ± 0.31
Thiamine biosynthesis protein ThiC	Cj0453	gi|121613325	−2.23 ± 0.38	−1.83 ± 0.32
ATP-dependent chaperone protein ClpB	Cj0509c	gi|121613623	−0.33 ± 0.15	−0.67 ± 0.15
2-oxoglutarate-acceptor oxidoreductase subunit OorD	Cj0535	gi|157414816	−0.87 ± 0.55	0.63 ± 0.66
2-oxoglutarate-acceptor oxidoreductase subunit OorA	Cj0536	gi|157414817	−0.73 ± 0.32	−0.67 ± 0.15
Aminohydrolase family protein, putative	Cj0556	gi|218562208	0.90 ± 0.57	1.13 ± 0.71
Quinol dehydrogenase membrane component NapH	Cj0782	gi|121612888	−0.67 ± 0.49	−0.55 ± 0.35
Bifunctional aconitate hydratase 2/2-methylisocitrate dehydratase	Cj0835c	gi|218562463	−1.00 ± 0.26	−0.93 ± 0.59
Thermonuclease family protein	Cj0979c	gi|121612563	−0.83 ± 0.38	−0.73 ± 0.68
Amino acid transporter, periplasmic solute-binding protein, putative	Cj0982c	gi|218562598	1.03 ± 0.38	0.77 ± 0.4
Arginyl-tRNA-protein transferase	Cj1035c	gi|121613462	−0.73 ± 0.29	−0.60 ± 0.26
Flippase	Cj1130c	gi|218562744	−0.27 ± 0.38	−0.67 ± 0.42
Cytochrome c553	Cj1153	gi|157415419	−0.77 ± 0.25	−0.53 ± 0.38
Cyclopropane fatty acid biosynthesis	Cj1183c	gi|57238055	1.07 ± 0.67	0.73 ± 0.32
NLPA family lipoprotein, putative	Cj1200	gi|218562812	0.47 ± 0.83	1.20 ± 0.56
Hemerythrin family non-heme iron protein	Cj1224	gi|121612625	−0.73 ± 0.25	−0.07 ± 0.21
Quinone-reactive Ni/Fe-hydrogenase, small subunit	Cj1267c	gi|157415531	−0.63 ± 0.46	−0.57 ± 0.21
Endoribonuclease L-PSP, putative	Cj1388	gi|218562997	0.77 ± 0.21	0.40 ± 0.36
Putative Ni/Fe-hydrogenase small subunit	Cj1399c	gi|218563003	0.73 ± 0.31	0.57 ± 0.15
Hypothetical protein C8J_1356 ATP/GTP-binding protein	Cj1450	gi|157415675	−0.87 ± 0.38	−0.80 ± 0.46
Transformation system protein CtsX	Cj1472	gi|121612126	−0.55 ± 0.7	−1.20 (single value)
Transformation system protein CtsP	Cj1473c	gi|121613067	−0.65 ± 0.07	−0.13 ± 0.31
Pyruvate-flavodoxin oxidoreductase	Cj1476c	gi|315124909	−0.87 ± 0.38	0.63 ± 0.42
Formate dehydrogenase, iron-sulfur subunit	Cj1510c	gi|157415732	−0.73 ± 0.71	−0.73 ± 0.45
Dephospho-CoA kinase	Cj1530	gi|218563123	0.73 ± 0.68	0.57 ± 0.74
Multidrug transporter membrane component/ATP-binding component	Cj1587c	gi|121613099	1.40 ± 0.53	1.00 ± 0.44
Two-component regulator (DNA binding response regulator), putative	Cj1608	gi|218563197	−0.07 ± 0.31	−2.50 ± 1.65
Citrate synthase	Cj1682c	gi|157415898	−0.80 ± 0.36	−0.73 ± 0.21
50S ribosomal protein L2	Cj1704c	gi|121612340	−0.47 ± 0.85	−0.80 ± 0.46
2-isopropylmalate synthase	Cj1719c	gi|121612265	0.83 ± 0.68	0.67 ± 0.21
Hypothetical protein CJE0598 CJIE2 prophage	CJE0598	gi|57238304	1.10 ± 0.28	0.77 ± 0.4
Flagellin	CJE1526	gi|57238729	−0.87 ± 0.25	−0.80 ± 0.44

## Discussion

Initial experiments describing the isolates characterized in this study [3,4] were performed before our laboratory had the capability of doing whole genome sequencing. Because attempts to create isogenic mutants for the CJIE1 prophage have so far been unsuccessful, the isolates chosen were therefore closely matched epidemiologically, by molecular typing results, and by initial microarray analysis (data not shown). We have recently obtained whole genome sequence data for the closed and finished genome (unpublished data). The gene content is the same for all four isolates. Of the 16 single nucleotide polymorphisms (SNPs) found outside homopolymeric tracts among in the four isolates only one was present in isolate 00–2426 and not in the other three isolates (unpublished data). This SNP results in the introduction of a stop codon that truncates the serine/threonine transporter (SstT) protein after 218 amino acids in isolate 00–2426 (unpublished data); the other three isolates should express the full-length protein. While little is known about the *C. jejuni* SstT protein, SstT in *E. coli* has been characterized in cloning, expression, and reconstitution experiments as a serine transporter [21] and, so far, no further activity has been associated with this protein. We detected expression of SstT (gi|121613737) only in Experiment 3 (Additional file 4), though it was detected in both isolates 00–2425 and 00–2426 and was up-regulated in the presence of bile salts. In the case of isolate 00–2426 it may have been the truncated version of the protein that was detected. Alternately, the colony that was subjected to whole genome sequencing may have carried a mutation within the gene encoding this protein. Serine utilization is an important carbon source that is also critical for host colonization [22]. Proteins SdaC (Cj1624c) and SdaA (1625c) have been associated with serine uptake, and SdaC appears to be sufficient to transport serine into the bacterial cell [22]. All four isolates used in this study were found to carry genes capable of expressing SdaC and SdaA. Expression of SdaA (gi|218563213) was detected in all four isolates, while SdaC was not detected in any of the four, suggesting it may have been produced in quantities too low to be recovered by our proteomics methodology. It seems doubtful that a truncation of SstT, if actually present, would result in the changes in protein expression seen in our studies, and is more likely that the differences in the presence/absence of CJIE1 were responsible.

Comparative proteomics with iTRAQ labelling was useful for demonstrating patterns of protein regulation in response to the presence of the CJIE1 prophage, to iron levels in the media, and to the presence of two different bile salt preparations in the media. Direct evidence for an effect of the CJIE1 prophage on expression of a small number of *C. jejuni* proteins was first obtained after growth of the organisms on medium containing blood, a finding confirmed in subsequent experiments using different growth conditions (MH and MH + SD). Effects on protein expression associated with the presence or absence of the CJIE1 prophage were therefore not due simply to growth on rich medium, but could be generalized to nutrient-limited media and medium containing 0.1% deoxycholate utilized previously to demonstrate induction of genes associated with *C. jejuni* virulence [15]. These results supported previous work indicating that the three CJIE1-carrying isolates exhibited greater adherence and invasion than the isolate without CJIE1 [3].

Additional evidence for regulation of protein expression associated with the presence or absence of CJIE1 was obtained by comparing the degree to which proteins were regulated after growth on MH as the reference condition, and MH + SD or MH + OX as the conditions used for comparison. Because the experimental design required these data to be obtained in two completely separate experiments involving either *C. jejuni* 00–2425 (CJIE1^+^) or *C. jejuni* 00–2426 (CJIE1^−^), it was not possible to quantify absolute expression levels, only the degree of change from the reference condition.

Changes in homopolymeric tract length could only be unambiguously associated with differences in quantitative protein detection/expression for two loci, Cj0685 (CipA) and for hypothetical protein Cj1305c/CJE1505. However, the length of homopolymeric tracts has been shown to change and are likely best expressed as the ratio of organisms in a population with homopolymeric tract length enabling full-length protein expression [23]. The data do suggest the homopolymeric tracts lengths determined by whole genome sequencing were determinative of the protein expression levels found in the proteomics experiments, further suggesting that the presence or absence of the CJIE1 prophage may somehow influence the homopolymeric tract length associated with some genes. This hypothesis requires further experimentation for verification.

### Proteins associated with changes in expression modified by CJIE1

The presence of CJIE1 affected the expression of 27 proteins when four isolates were grown on MH + blood, 19 of which were not encoded by the CJIE1 prophage (Table 1). Growth of two isolates (+/−CJIE1) on MH and MH + SD resulted in differential expression of 27 proteins, of which 11 were also observed during growth on MH + blood. Only two prophage proteins were expressed on MH+/−SD, suggesting that the expression of phage structural proteins may be more favored on rich medium. It is also possible that the proteins showing differential expression on only one or the other medium, 10 of which were unique to MH + blood and 16 of which were unique to MH and MH + SD, exhibited this characteristic because of regulatory pathways specific for each growth medium. This should be a good subject for further directed studies.

The proteins regulated by CJIE1 when isolates were grown on MH + blood did not appear to be a random subset of the total proteins detected, either in terms of function or chromosomal location. Two of the up-regulated proteins and three of the down-regulated proteins (total = 5/21 or 24%; Table 2) were in some way associated with O-linked glycosylation (Cj1305c, Cj1310c), capsule biosynthesis (Cj1426c), or other carbohydrate synthesis (Cj0288c, Cj0685c). Cj1426 was previously identified as one of the genes regulated by CosR [24]. Though the co-localization of genes encoding Cj1098-Cj1101 was striking, the differences in protein function of the gene products would suggest that these genes are not part of an operon. Protein expression in the presence of bile salts, compared with growth on MH + blood, was also different for expression of CJ1098 and Cj1101 compared with CJ1099, indicating that protein expression was not co-regulated. No obvious genomic elements that could affect transcription were noted in the region upstream of Cj1098 in the re-annotation of the NCTC 11168 genome (NC_002163). Further work is required to verify the protein expression observations seen here and to elucidate the mechanisms by which they occur.

Each of the nine proteins exhibiting differences of expression associated with the presence or absence of the prophage on all three media appeared to have important biological functions in *C. jejuni*. The invasion phenotype protein (CipA) was expressed in greater amounts when CJIE1 was present, and was further up-regulated approximately two-fold by the presence of bile salts. CipA (Cj0685c) has been annotated as a putative sugar transferase with homology to proteins within the capsule synthesis locus [25]. Transposon mutagenesis of the gene encoding this protein reduced invasion of INT-407 and Caco-2 cells to about 2% of wild type levels though there was minimal change in motility [26]. Major protein sequence differences in CipA were associated with loss of the capacity of the strain to invade Caco-2 cells [27]. The increased expression of this protein in isolates carrying the CJIE1 prophage might therefore be at least partly responsible for the increased invasion of INT-407 cells found in earlier work [3].

Cj1429 was also highly up-regulated on MH + blood in the CJIE1^+^ isolate (Table 1). This protein was also induced quite strongly by 0.1% sodium deoxycholate or 2.5% Oxgall, though the effect was not as pronounced in isolate 00–2425 carrying CJIE1. Cj1429 expression therefore appears to be part of the general bile stress response. It is annotated as a hypothetical protein, and BLAST searches provide no further clues as to its function. The gene encoding Cj1429 is variably present in the capsular polysaccharide biosynthesis locus [28]; it therefore constitutes a strain-specific protein that may or may not be associated with virulence. It is possible that, along with CipA, this protein may modify the sugar composition of the capsule to increase adherence and invasion of *C. jejuni* isolates. Capsular polysaccharide is associated with reduced surface hydrophobicity, increased serum resistance, increased invasion in cell culture, and increased virulence in a ferret model [29]; expression or overexpression of Cj1429 might alter these properties. Resequencing of *C. jejuni* NCTC11168 after serial passage in mice detected changes at several loci associated with homopolymeric tracts. The gene encoding Cj1429 was one of the loci showing significant enrichment after passage for variants in which the gene was in-frame and therefore expressed [23]. This further implicates Cj1429 as a key virulence factor.

A methyltransferase homologous to Cj1426 in *C. jejuni* NCTC11168 was also part of the capsule locus and was differentially expressed in the presence and absence of the CJIE1 prophage. This methyltransferase is responsible for adding a 6-*O*-Me residue onto the heptose of the capsular polysaccharide repeat unit [30].

Acetate kinase and phosphate acetyltransferase are key enzymes responsible for production of acetyl CoA, a key molecule in metabolism. Acetate kinase produces acetyl phosphate, which in *E. coli* is a global regulator affecting capsule biosynthesis, biofilm development, pathogenicity, flagellar biosynthesis, pilus assembly, nitrogen assimilation, and osmoregulation [31]. It was annotated as being capable of acetylating CheY, thereby increasing signal strength during flagellar rotation (see accession number NC_002163). Acetate kinase and phosphate acetyltransferase were the second and third most highly expressed CJIE1-regulated proteins on MH + blood, respectively. Compared with the reference condition (growth on MH), growth of isolates on MH + SD resulted in down-regulation of acetate kinase to approximately the same extent in isolates +/− CJIE1 (Table 5); this effect was much smaller on MH + OX. Despite the fact that both sodium deoxycholate and Oxgall are bile salts, it would appear the differences in composition were sufficient to elicit a slightly different regulatory response. Furthermore, the presence or absence of the CJIE1 prophage did not appear to have much effect on the decrease in expression over the reference condition (growth on MH). Phosphate acetyltransferase was expressed approximately 5-fold higher in isolate 00–2425 compared to isolate 00–2426 when both isolates were grown on MH + blood, MH, and MH + SD. In this case, however, the expression of the protein was increased 2 – 4 fold only in the absence of CJIE1 when grown on bile salts (Table 5) compared with growth on MH. The presence of CJIE1 appeared to abrogate the response of phosphate acetyltransferase expression to bile salts. Though associated with a common biochemical pathway, acetate kinase and phosphate acetyltransferase appear to be regulated quite differently. While little is known about the roles of these proteins in *C. jejuni*, they may be key regulators of a number of cellular responses, and are possible candidates for regulation of the general bile response described below. Though the genes encoding these two proteins are contiguous in the *C. jejuni* chromosome, the regulation of protein expression appeared to be quite different under the different conditions used.

Phosphate acetyltransferase and FliS have both been implicated in biofilm production due to the observation that mutants in the genes encoding each of these proteins eliminated floc formation and pellicle formation [32]. However, neither of these proteins was more highly expressed in *C. jejuni* isolates from biofilms or planktonic cells [33]. The differences in FliS expression in these studies were not significant, and it is not clear that phosphate acetyltransferase was co-regulated with this protein.

The aspartate carbamoyltransferase catalytic subunit (PyrB) was expressed at higher levels in strain 00–2425. This subunit is part of a multimeric enzyme complex that catalyzes the first step in the pyrimidine biosynthesis pathway, and controls the rate of pyrimidine biosynthesis by feedback inhibition [34,35]. Changing the expression of only the catalytic PyrB peptide could change the stoichiometry of the catalytic and regulatory sites, but it is not clear what the biological effect on the bacterial cell might be.

Recently, oligoendopeptidase F (PepF) has been characterized as a signal peptidase required for protein secretion [36]. It has been associated with changes in peptidoglycan structure when bacteria were grown in rich medium, and plays a role in pyruvate metabolism, including acetate production. It is possible, but speculative, that increases in expression of PepF could result in concomitant changes in protein secretion, and that this may also require the activity of acetate kinase and phosphate acetyltransferase to restore acetate homeostasis.

The ATP-dependent DNA-helicase UvrD functions to carry out DNA excision repair in stalled, regressed replication forks [37]. When this protein is overexpressed, the frequencies of recombination subsequent to conjugation or transformation are reduced, while sensitivity to ultraviolet light is increased [38], likely due to the fact that in E. coli UvrD displaces RecA from DNA [39] and mutations in *uvrD* induce the SOS response [40]. Among other things, we speculate that these properties could suggest that overexpression of UvrD might also suppress the induction of prophages.

### Expression of prophage proteins, effects of CJIE1 prophage on expression, and induction of prophages by growth on bile salts

Expression of a limited number of prophage proteins was detected in this study. As expected, proteins encoded by CJIE1 genes were detected only in isolate 00–2425, while isolate 00–2426 did not express these proteins (Table 2); these observations provide support for the validity of results and the experimental design. When grown on MH + blood, both the CJIE1 and CJIE4 prophages were expressed at low levels, as shown by detection of a relatively few prophage structural proteins. Only two CJIE1 prophage proteins were expressed on MH (Table 2), and one was a cargo gene. Presumably these two proteins were the ones most highly expressed from the prophages. Growth on either MH + SD or MH + OX resulted in unchanged levels of the CJIE1 repressor protein CJE0215, but increased expression of a second repressor protein, the signal peptidase I adjacent to *panB* within the 00–2425 genome (Additional file 2: Table S2). An additional prophage structural protein, bacteriophage transposition protein B (CJE0269), was also detected in this experiment and was more highly expressed on media containing bile salts, consistent with induction of the prophage by bile salts. Malik-Kale and colleagues [15] also detected transcriptional up-regulation of several CJIE1 proteins in clinical *C. jejuni* isolate F38011 upon growth in 0.1% sodium deoxycholate. These included the phage structural protein for tail fiber H (CJE0230), a muramoyl-pentapeptide carboxypeptidase (CJE0241), two hypothetical proteins (CJE0230 and CJE0243) and DNA adenine methylase (CJE0220) (see [4] for updated annotation). While different loci were detected than in our current work, these results were also consistent with induction of CJIE1 by bile salts.

ORF11, a unique protein encoded by a cargo gene in a subset of *C. jejuni* isolates [3,4], and the extracellular DNase associated with loss of natural transformation (CJE0256) were the only other CJIE1 proteins with detectable expression in this series of proteomics experiments. The DNase was downregulated by growth in both bile salt preparations. ORF 11 was not affected by changes in iron content of the medium but was induced in the presence of bile salts. The levels of induction of ORF11 were roughly similar to those of DNA transposition protein B, so that it is not clear whether ORF11 protein expression was specifically induced by bile salts or whether the increased expression was caused by induction of the prophage with a resulting increase in copy number of the gene. The fact that this protein was one of only a few CJIE1 proteins that could be detected in all proteomics experiments suggests it may be responsible for the biological effects of the prophage [3].

There was clear induction of CJIE4 prophage genes for at least some structural and functional proteins upon growth on both MH + SD and MH + OX as demonstrated by the down-regulation of the phage repressor, CJE1429, and upregulation of the recombinase (CJE1418) and capsid (CJE1458) proteins (Additional file 2: Table S2). Because of the absence of demonstrable quantities of other prophage proteins, including tail proteins, it is not clear that infectious phage particles were produced. The second repressor, Cj1440, exhibited slightly increased expression only in the presence of 2.5% Oxgall in isolate CJIE1^−^ 00–2426. CJIE4 capsid protein expression was lower the presence of CJIE1 than in its absence, suggesting that the presence of CJIE1 may decrease CJIE4 induction. The expression of two genes encoding the CJIE4 toxin-antitoxin protein (CJE1470) and phage terminase, small subunit protein (CJE1472) (see [41]) was also found to be up-regulated when *C. jejuni* isolate F38011 was grown on 0.1% sodium deoxycholate [15], further supporting the hypothesis that this prophage is induced by growth of the bacterium on 0.1% sodium deoxycholate.

Other CJIE4 proteins with altered expression in bile salts included the extracellular endonuclease and three hypothetical proteins (Additional file 2: Table S2). Two of these (CJE1439 and CJE1441) were within a region of variability - an indel - within the CJIE4 genome (manuscript in preparation). CJE1466 was consistently expressed in all proteomics experiments, even in the absence of evidence for prophage induction, suggesting it may also be the product of a cargo gene. The DNA/RNA non-specific endonuclease (CJE1441) from CJIE4 was the only protein from this prophage with decreased expression in the presence of bile salts, exhibiting a 4-fold decrease in the presence of 0.1% sodium deoxycholate and a 7 – 7.5-fold decrease in the presence of 2.5% Oxgall, (Additional file 2: Table S2). This endonuclease, like the CJIE1 DNase, is a non-essential protein affecting DNA-uptake and natural transformation [2]. The expression of both proteins may not be highly adaptive under conditions of bile stress.

### Growth of *C. jejuni* on medium containing 0.1% sodium deoxycholate or 2.5% Oxgall provides evidence of an adaptive bile response involving a majority of the proteome

Malik-Kale et al. [15] previously suggested that the presence of 0.1% sodium deoxycholate was associated with the induction of virulence genes and a general bile stress response. Fox et al. [16] also demonstrated a response to 2.5% ox-bile involving about 48 proteins. The results presented here support the existence of a more general stress response involving a large proportion of *C. jejuni* proteins. Down-regulation in the presence of 0.1% deoxycholate was observed for most or all proteins associated with energy production and conversion, amino acid transport and metabolism, nucleotide transport and metabolism, carbohydrate transport and metabolism, coenzyme transport and metabolism, lipid transport and metabolism, transcription, post-translational modification, protein turnover, chaperones, inorganic ion transport and metabolism, and secondary metabolites biosynthesis, transport, and catabolism. Most proteins associated with cell motility were up-regulated in the presence of 0.1% sodium deoxycholate, as were many within COGs associated with cell wall/membrane/envelope biogenesis and replication, recombination, and repair. CmeA, CmeB, and CmeC expression was found to be increased in both this work and by Malik-Kale et al. [15]. In addition, the expression of proteins associated with the presence and induction of CJIE1 and CJIE2 was increased in both this work and that described earlier [15].

There were some consistent patterns of protein regulation known to be associated with virulence, further supporting the conclusions of Malik-Kale et al. [15]. Proteins involved in capsule synthesis and transport were up-regulated in the presence of bile salts, as was the flippase involved in N-linked glycosylation and flagellar structural genes and biosynthesis regulation. These proteins included FlhA and FlhF. FlhA plays an important role in flagella formation, motility and invasion by regulating the expression of σ^28^- and σ^54^-regulated genes [7], and could other key genes as well. It is also part of the flagellar type three secretion system and the flagellar MS ring [42].

The strong and consistent down-regulation of CiaB in the presence of both bile salts was surprising. Malik-Kale et al. [15] conducted a series of transcriptomic experiments that strongly indicated expression of the *ciaB* gene was up-regulated in the presence of 0.1% sodium deoxycholate in Mueller-Hinton agar, precisely the same conditions used in the current study. Such expression differences between transcriptomic and proteomic experiments may be attributed to post-transcriptional regulation [9]. Malik-Kale et al. [15] also found that CiaB was present in the supernatants of *C. jejuni* prepared from MH + SD plates, suggesting that our observation of less cellular CiaB in isolated bacteria could have resulted from export of the protein out of the bacterial cell.

### Iron regulated proteins

All iron acquisition proteins and all oxidative stress proteins showing differences in expression on MH medium compared with MH + blood have been previously described and the roles defined [43-45]. Every protein associated with the putative siderophore-uptake system (Cj1658 – Cj1663 showed higher expression on MH medium than on MH + blood, and all 5 transferrins (Cj173-175, 177, and 178) were up-regulated under conditions with decreased iron, as has previously been shown [44]. Cj177 and Cj178 were two of the three iron-regulated proteins showing the highest levels of up-regulation under lower iron concentrations. Of the five proteins associated with the ferric enterochelin-uptake system (Cj0755, Cj1352-1355), three were more highly expressed at lower iron concentrations. These were the ferric receptor CfrA (Cj0755), the enterochelin uptake ATP-binding protein CeuD (Cj1354), and the ABC transporter CeuE (Cj1355) [45]. The two proteins that form the inner membrane permease, CeuB (Cj1352) and CeuC (Cj1353) did not exhibit changes in expression levels when isolates were grown on MH + blood compared with MH agar. Together these observations suggest that the number of permease molecules may not be the limiting factor for uptake of iron through the ferric enterochelin-uptake system.

Increased protein expression associated with growth in lower iron concentrations was observed for all 5 proteins associated with the hemin-uptake system, which are all part of an operon, *chuABCD* (Cj1614-17) [44,45].

Additional known iron uptake proteins that did not demonstrate obvious regulation in the current study included a putative lipoprotein (Cj0176c), FeoA, FeoB, and a number of the proteins involved with iron-associated energy transduction systems [45]. Despite its close genetic linkage to the genes encoding the transferrins, the gene encoding Cj0176c is not a part of the two operons that include the genes encoding the transferrins [46]; this is consistent with the differences in expression seen here. Palyada and colleagues [47] previously observed that no significant differences in expression of FeoA and FeoB were seen upon changing the level of ferrous iron in the medium, which was taken as verification that FeoB is not required for ferrous iron uptake in *C. jejuni*[48]. It was of interest that only three (ExbB1, Cj0179; ExbD2, Cj1629; TonB, Cj0753) of the nine proteins known to be associated with energy transduction systems showed changes in expression resulting from changes in iron content in the growth medium. It is possible that TonB may function with any of the ExbBD uptake systems [45]. Despite being encoded by genetically unlinked loci, perhaps ExbB1 and ExbD2 associate to form a functional energy transduction system as well. Importantly, Palyada and colleagues [47] found that proteins associated with all three Exb energy transduction systems showed changes in protein expression associated with differences in iron availability in transcriptomic experiments. The reasons for the different pattern of expression seen for these specific proteins in the current study are not clear and require further investigation.

Iron regulation and the oxidative stress response are tightly linked [43,46]. The oxidative stress response responds to iron released by oxidative destruction of iron-sulfur centers in proteins [42] and is responsive to iron levels in the bacterium’s environment. As expected catalase, the antioxidant AhpC protein, and two thioredoxins exhibited higher levels of expression at lower levels of iron in the growth medium [45,47]. MsrA (Cj0637c) was also up-regulated in conditions of lower iron concentration, but only in the *C. jejuni* 00–2425 isolate carrying CJIE1. Methionine sulfoxide reductases reverse the oxidative destruction of methionine residues by catalyzing the conversion of methionine sulfoxides back to methionines, utilizing thioredoxin(s) as the electron donor [45]. The presence of CJIE1 might provide an adaptive function under conditions of oxidative stress not present in isolates without the prophage. Three methionine biosynthesis proteins were up-regulated in medium with lower iron concentrations (Table 6); this could be part of the response to the destruction of methionines by oxidation under conditions of oxidative stress. Homoserine serves as a precursor for both methionine and S-adenosyl methionine (SAM) biosynthesis. SAM is a major methyl donor in cell metabolism [49]; this could provide another functional rationale for up-regulation of proteins involved in methionine biosynthesis.

Ferredoxin expression was decreased when isolates were grown on MH medium without blood (Table 6), consistent with earlier data showing that this protein is induced in the presence of iron [47,50]. Ferredoxin has been classified as a protein involved in electron transport [44]; it may also be involved in the oxidative stress response [47,50]. Additional proteins found to be regulated by iron in the current study have also been detected in earlier studies, namely ThiC [47] and Cj1587 [44].

The results of the comparative proteomics experiments presented here corresponded to what was already known about the response to iron in *C. jejuni*. This validates the methods and experimental design, and strongly supports the credibility of the other data collected in these experiments. Furthermore, these observations suggest that proteins that did not appear to be regulated in a statistically significant manner may still have biologically relevant changes in expression.

A number of other iron-regulated proteins were identified as such for the first time in this study. MetE (Cj1201), Cj1200 are proteins regulated by LuxS/AI-2 (autoinducer 2) in the presence of hydrogen peroxide (oxidative stress) [49]. Cj0425, a putative periplasmic protein identified in bioinformatics analysis as having a high probability of association with oxygen tolerance [51], was up-regulated by decreased iron only in the presence of CJIE1 and expressed at lower levels in the presence of bile salts. An ATP-dependent CLP protease ATP-binding subunit was identified as well in this study as part of the stress response; a similar protein was detected as being subject to iron regulation in the current study (Table 6), but was annotated as being similar to homologs from a number of *Treponema* isolates. This protein may, in fact, be part of the oxidative stress response.

## Conclusions

The presence of the CJIE1 prophage appeared to selectively affect the expression of a small proportion of virulence-associated proteins of *C. jejuni*. This could be of benefit for the *C. jejuni* bacterium by facilitating adaptation to immediate environmental changes as the bacterium traverses the gut of the infected human and preadaptation to the niche in which the organism is capable of invading host cells and tissues. There was also a general bile response involving a majority of protein expressed by the organism. The presence of bile salts appears to be a signal for induction of both CJIE1 and CJIE4 prophages. Prophage induction within the gut could result in transmission of the temperate phage to new bacterial hosts or the creation of diversity within the *C. jejuni* population through movement and integration of the prophage into new chromosomal locations or the generation of large chromosomal rearrangements [52].

## Methods

### Isolates and growth conditions

The isolates used were chosen because they were all outbreak type 1 from the large waterborne outbreak in Walkerton, ON, Canada in 2000 [53] and the genomes were very similar in microarray, Southern blotting, and PCR experiments, except for the presence (isolates 00–2425, 00–2538, and 00–2544) and absence (00–2426) of the CJIE1 prophage [3,53]. These findings were generally confirmed by the closed and finished whole genome sequence data (data not shown). Of the 16 single nucleotide polymorphisms (SNPs) found outside homopolymeric tracts among all 4 isolates, only one was present in isolate 00–2426 and not in the other 3 isolates (data not shown). This SNP results in the introduction of a stop codon that truncates the serine/threonine transporter (SstT) protein after 218 amino acids (data not shown). These data will be included in a future publication. In addition, a large plasmid was detected in isolate 00–2544. Accession numbers for whole genome sequences are 00–2425, CP006729; 00–2426, CP006708; 00–2538, CP006707; 00–2544, CP006709 (genome) and CP006710 (plasmid). CJIE1 was inserted between *panB* (Cj0298c homolog) and a gene encoding beta-lactamase (Cj0299 homolog) in isolates 00–2425 and 00–2538, and into the gene encoding the McrB restriction endonuclease McrB subunit (homolog of Cj0139) in isolate 00–2544 [unpublished data]. The use of three isolates carrying CJIE1 therefore provided a control for the position of the prophage in the chromosome. Three prophage-carrying isolates were originally also used as a strategy to minimize any possible effect(s) due any undetected genetic or DNA sequence changes such as SNPs, since whole genome sequence data for these isolates has only been obtained very recently. Comparisons of the three isolates carrying CJIE1 with the isolate lacking this prophage have previously shown differences in adherence and invasion [3].

*C. jejuni* isolates were routinely grown on Oxoid Mueller-Hinton agar (Oxoid Inc., Nepean, ON) containing 10% sheep red blood cells (MH + blood) for 48 – 72 h at 37°C under microaerobic atmosphere (5% O_2_, 10% CO_2_, 85% N_2_). Further investigations were aimed at determining whether any changes in protein expression due to the presence or absence of the CJIE1 prophage could be replicated under conditions thought to induce virulence genes, as well as whether any such differences were apparent only in the presence of sodium deoxycholate or in the presence of bile salts in general. To this end isolates were also grown on Mueller-Hinton agar (MH), MH containing 0.1% sodium deoxycholate (Sigma-Aldrich), designated MH + SD (or SD where space is restricted), and MH containing 2.5% BD Difco Oxgall (BD Biosciences, Mississauga, ON), designated MH + OX (or OX where space is restricted). Storage was in either 20% skim milk or glycerol peptone water (25% v/v glycerol, 10 g/L neopeptone, 5 g/L NaCl) at -80°C.

### Crude protein preparation from whole bacterial cells

The proteomics methods used were based partly on those previously published by Wiśniewski et al. [54]. *C. jejuni* were grown microaerobically on at least three plates of each culture medium for 48 h at 37°C; more plates were required for cultures on MH + OX due to the significant growth inhibition of the Oxgall. Bacteria on plates were suspended in 1 ml sterile Dulbecco’s PBS, pH 7.0 – 7.2 (Gibco; Invitrogen) using a sterile spreader; fractions from different plates were collected into a sterile 15 ml centrifuge tube. After centrifugation for 15 min at 1455 × g the bacterial pellets were suspended in fresh PBS, and the contents of the tube were transferred to a 1.5 ml microfuge tube. This suspension was centrifuged at 16,000 × g in a microfuge and the PBS was removed. At this point the sample was either used for the next step of the protein preparation protocol or frozen for up to 2 weeks at -20°C.

A 250 μl volume of sterile 18 MΩ (MilliQ) water was added to the pelleted bacteria. About 100 μl of acid-washed 212 – 300 μm glass beads (Sigma-Aldrich Canada Ltd., Oakville, ON) was added to each tube and the bacteria were resuspended by vortexing for 10 s at the highest setting. The resulting suspensions were boiled for 5 min in a boiling water bath. Another 250 μl of sterile MilliQ water was added and the suspensions were lysed by incubating for 5 min at low speed on a Genie 2 vortex mixer fitted with a 12 place Ambion Vortexer Adapter for Genie 2 Vortex Mixer attachment (Applied Biosystems Canada, Mississauga, ON); this constituted a “bead-beating” step. After centrifugation for 1 min at 3000 rpm (664 × g) in a microfuge the cloudy, protein-containing supernatant was collected into a sterile 15 ml centrifuge tube. Addition of 500 μl MilliQ water, vortexing, bead-beating, centrifugation, and collection of supernatant were repeated until the supernatant was clear. Protein preparations were used immediately or stored at -80°C for up to two months.

The protein concentration of each preparation was estimated using a Pierce Protein Assay kit (Fisher Scientific, Whitby, ON) according to the manufacturer’s protocol.

### Protein modification and digestion

Crude protein suspensions containing 100 μg protein were placed in 1.5 ml microfuge tubes and dried without heating in a Savant DNA120 SpeedVac Concentrator (Fisher Scientific). Each protein preparation was suspended in 50 μl of freshly made SDS solubilization buffer (4% SDS, 50 mM HEPES buffer pH 8.3, 100 mM DTT), heated at 95°C for 5 min, and placed in a -20°C freezer overnight. The following morning Nanosep 10 K cartridges (VWR International LLC, Mississauga, ON) were prepared by first adding 200 μl water to the cartridge and centrifuging at 10,000 × g for 10 min, then by adding 200 μl of fresh Urea Exchange Buffer (UEB; 8 M urea in 50 mM HEPES, pH 8.3) and centrifuging at 10,000 × g until all liquid was removed from the cartridge.

A 7-fold volume (350 μl) of UEB was added to each 50 μl of protein in SDS solubilization buffer. Samples were then placed into the prepared Nanosep spin filter cartridges and centrifuged at 10,000 × g. After removing the flow-through, samples were washed twice with UEB, each time centrifuging to remove almost all liquid in the cartridge and discarding the flow-through.

Proteins were then alkylated by adding 100 μl of 50 mM iodoacetamide (IAA Reagent, Sigma-Aldrich) in UEB and shaking for 5 min at RT on an MBI Thermo-Shaker (Montreal Biotech, Kirkland, PQ, Canada) covered with tin foil, followed by a further 20 min incubation without shaking in the dark, followed by centrifugation at 10,000 × g for approximately 10 min. The spin filter was washed three times with UEB, centrifuging and discarding the flow-through as above each time to remove almost all liquid. The buffer was then exchanged by washing twice with 150 μl of 50 mM HEPES, pH 8.3, centrifuging and discarding the flow-through as above each time to remove almost all liquid.

DNA was removed by the addition of 50 μl of freshly made Benzonase (Sigma-Aldrich) solution (20 U/μl Benzonase in 42 mM HEPES, pH 8.3 containing 2 mM MgCl_2_) to the membrane followed by mixing at 600 rpm for 2 min at RT on an MBI Thermo-Shaker and incubation for 30 min at RT. After this incubation, the cartridge was washed three times with 100 μl of 50 mM HEPES, pH 8.3, centrifuging and discarding the flow-through as above each time to remove almost all liquid.

One vial (100 μg) of trypsin (Trypsin Gold, mass spectrometry grade, Promega ) was previously dissolved in 100 μl of 0.1% formic acid (vol/vol), distributed in 5 μl (5 μg), and kept frozen at -20°C. To prepare fresh solution just before use for trypsinization of proteins, 45 μl of 50 mM HEPES, pH 8.3 was added to a 5 μl (5 μg) trypsin for each sample to be trypsinized. This was added to each cartridge, mixed for 1 min at 600 rpm at RT on an MBI Thermo-Shaker followed by incubation ON min at 37°C in a humidified atmosphere. The next day peptides were recovered from the cartridge. First 50 μl of 50 mM HEPES, pH 8.3 was to the 50 μl trypsin digest in the cartridge and mixed at 600 rpm for 3 min at RT. The cartridge was then inverted and placed into a fresh microfuge collection tube, followed by centrifugation in the microfuge at 10,000 × g for 1 min. The addition of buffer to the cartridge and subsequent centrifugation was done twice more to ensure optimal peptide yield, resulting in a final 200 μl volume. The pooled peptide solution was frozen at -20°C until peptides were either labelled with iTRAQ reagents or used as unlabelled peptide preparations in liquid chromatography and mass spectrometry.

### iTRAQ labelling

Peptides were first dried without heating in a Savant DNA120 SpeedVac Concentrator, then suspended in 30 μl 100 mM HEPES, pH 8.3. 70 μl 100% ethanol was added to each iTRAQ label and the resulting solution was mixed by vortexing at high speed for 30 s followed by vortexing at medium speed for 30 min in a foam vortexer adapter. Each iTRAQ label solution was then added to a peptide mixture, vortexed 20 s at the highest setting, collected by centrifugation in a microfuge, and incubated ON at RT. The next day 150 μl sterile MilliQ water was added to quench the labelling reaction and the mixtures were dried in the SpeedVac concentrator. These mixtures were frequently stored at -20°C until the next step.

Labelled peptides were thawed, dissolved in 40 μl water, vortexed for 30 min on medium, and centrifuged in a microfuge for 2 min at 16,000 × g to remove insoluble material. After removal of supernatant to a fresh tube, 1 μl of each labelled peptide mix was added to 56 μl nano LC buffer A (2% acetonitrile, 0.1% formic acid) in a 300 μl PTFE vial. These pre-scans were subject to separation by nano liquid chromatography as outlined below and the results were used to normalize the amount of each labelled peptide mixture for chromatography and mass spectrometry. Labelled peptide fractions were mixed to provide the same amount of peptide using approximately 10 μl volumes of each labelled peptide.

### Liquid chromatography and mass spectrometry

iTRAQ-labelled tryptic peptide samples (100 μg) were fractionated by high-pH, C_18_-reversed phase liquid chromatography on a micro-flow Agilent 1100/1200 series system (Agilent Technologies), using a Waters XBridge C_18_ guard column (10 mm long, 2.1 mm inner diameter, 3.5 μm particles) and a Waters XBridge C_18_ analytical column (10 cm long, 2.1 mm inner diameter, 3.5 μm particles). Mixed peptides were dried and suspended in LC buffer A (20 mM ammonium formate, pH 10), then resolved by a gradient of LC buffer A and buffer B (20 mM ammonium formate and 90% acetonitrile, pH 10). The gradient started at 3% B from 0–10 min, 8-11% B from 10–17 min; 11-60% B from 17–75 min; 95% B from 75–80 min; and 3% B from 80–170 min at a constant flow rate of 150 μl/min. Fractions were collected across the peptides elution profile (10–75 min). Fractions were dried and resuspended in 40 μl of nano LC buffer A.

Each fraction was separately analysed using a nano-flow Easy nLC II (Thermo Fisher Scientific) connected in-line to an LTQ Orbitrap Velos mass spectrometer (Thermo Fisher Scientific) with a nanoelectrospray ion source (Thermo Fisher Scientific). The peptide fractions (5 μl) were loaded onto a C_18_-reversed phase trap column (2 cm long, 100 μm inner diameter, 5 μm particles) with 100% buffer A (2% acetonitrile, 0.1% formic acid) at 4 μl/min for a total volume of 30 μl, and then separated on a C_18_-reversed phase column (10 cm long, 75 μm inner diameter, 3 μm particles). Both columns were packed in-house with ReproSil-Pur C_18_-AQ resin (Dr. Maisch). Peptides were eluted using a linear gradient of 0-30% buffer B (98% acetonitrile, 0.1% formic acid) over 120 min at a constant flow rate of 300 nl/min. Total LC/MS/MS run-time was 160 minutes, including the loading, linear gradient, column wash at 95% buffer B, and the equilibration.

Data were acquired using a data-dependent method, dynamically choosing the top 10 abundant precursor ions from each survey scan for isolation in the LTQ and fragmentation by HCD at 45% normalized collision energy. The survey scans were acquired in the Orbitrap over *m/z* 300–1700 with a target resolution of 60,000 at *m/z* 400, and the subsequent fragment ion scans were acquired in the Orbitrap over a dynamic *m/z* range with a target resolution of 7500 at *m/z* 400. The lower threshold for selecting a precursor ion for fragmentation was 1000 counts. Dynamic exclusion was enabled using a list size of 500 features, a *m/z* tolerance of 15 ppm, a repeat count of 1, a repeat duration of 30 s, and an exclusion duration of 15 s, with early expiration disabled.

### Data processing

All spectra were processed using Mascot Distiller v2.3.2 (Matrix Science), and database searching was done with Mascot v2.3 (Matrix Science). Searches were performed against an in-house built, non-redundant database consisting of NCBI’s Genome database of bacteria [ftp://ftp.ncbi.nlm.nih.gov/genomes/Bacteria/] and prophage sequences generated in-house. The decoy database option was selected and the following parameters were used: carbamidomethylation (C) and iTRAQ (K and N-terminus) as fixed modifications, oxidations (M) as a variable modification, fragment ion mass tolerance of 0.5 Da, parent ion tolerance of 10 ppm, and trypsin enzyme with up to 1 missed cleavage. Mascot search results were imported into Scaffold Q + v3.4 (Proteome Software) and filtered using 80% confidence for peptides, 99% confidences for proteins, and at least 2 peptides per protein.

### PCR and DNA sequencing for determining the presence and sequence integrity of genes encoding highly downregulated proteins in isolate 00–2426 without CJIE1

To confirm whether selected genes encoding selected proteins had changes in their promoters or coding regions PCR was done using the primers and conditions summarized in Additional file 2: Table S1 and using methods described previously [4]. The MgCl_2_ concentration was 2.0 mM in all cases.

### Data analysis for figure

Data from three biological replicates of each strains (2425 and 2426) grown in 4 media (MH, MH + blood, SD and OX) was exported from Scaffold v.3.4.5. (1) and formatted in Excel. GI numbers and Log2 fold-changes were imported in DanteR v.0.1.1. (available from [http://omics.pnl.gov/software/DanteR.php]) for cluster analysis. For each strain, two factors (Replicate and Media) were assigned to the data table. Hierarchical clustering was performed on columns and rows using Euclidean distance and the complete linkage agglomeration method.

### Availability of supporting data

Some of the supporting data have been included as Additional files 1, 3, and 4. These files represent the Excel Samples Report created by the Scaffold software after removal of entries for which there was no log_2_ change values and after calculating means for each group of data. Accession numbers for whole genome sequences are 00–2425, CP006729; 00–2426, CP006708; 00–2538, CP006707; 00–2544, CP006709 (genome) and CP006710 (plasmid).

The mass spectrometry proteomics data have been deposited to the ProteomeXchange Consortium (http://www.proteomexchange.org) via the PRIDE partner repository [55] with the dataset identifiers PXD000798, PXD000799, PXD000800, and PXD000801.

Data for experiment 1 have been deposited under the ProteomeXchange title “Effects of *Campylobacter jejuni* CJIE1 prophage on protein expression experiment 1” with the dataset identifier PXD000798. The iTRAQ labels for this experiment (all three replicates) were for *C. jejuni* isolate: 00–2425, 114; 00–2426, 115; 00–2538, 116, 00–2544, 117.

Data for experiment 2 have been deposited under the ProteomeXchange title “Effects of *Campylobacter jejuni* CJIE1 prophage on protein expression experiment 2” with the dataset identifier PXD000799. The iTRAQ labels for this experiment were different for each replicate experiment as in the following description. Replicate Y1: 00–2425 grown on MH agar, 114; 00–2425 grown on MH + SD, 115; 00–2426 grown on MH agar, 116; 00–2426 grown on MH + SD, 117. Replicate Y2: 00–2425 grown on MH agar, 115; 00–2425 grown on MH + SD, 116; 00–2426 grown on MH agar, 117; 00–2426 grown on MH + SD, 114. Replicate Y3: 00–2425 grown on MH agar, 116; 00–2425 grown on MH + SD, 117; 00–2426 grown on MH agar, 114; 00–2426 grown on MH + SD, 115.

Data for experiment 3 have been deposited under the ProteomeXchange title “Effects of *Campylobacter jejuni* CJIE1 prophage on protein expression experiment 3” with the dataset identifier PXD000800. The iTRAQ labels for this experiment (all three replicates) were for *C. jejuni* isolate 00–2425 grown on: MH + blood, 114; MH agar, 115; MH + SD, 116; MH + OX, 117.

Data for experiment 4 have been deposited under the ProteomeXchange title “Effects of *Campylobacter jejuni* CJIE1 prophage on protein expression experiment 4” with the dataset identifier PXD000801. The iTRAQ labels for this experiment (all three replicates) were for *C. jejuni* isolate 00–2426 grown on: MH + blood, 114; MH agar, 115; MH + SD, 116; MH + OX, 117.

## Competing interests

The authors declare that they have no competing interests.

## Authors’ contributions

Conceived and designed the work: CGC. Performed laboratory experiments: CGC, PC, SJM, KC, DML, KN. Performed Mascot and Scaffold analysis: GRW. Bioinformatics analysis and annotation of whole genome sequence: MW, CGC. Performed statistical analysis and figure: CGC, PS. Performed all other data analysis: CGC. Wrote the manuscript: CGC. Provided assistance with funding, acquisition of additional supplies and a Co-op student (KN), and valuable advice for the written manuscript: MWG. All authors have read and approved the final manuscript.

## Supplementary Material

Additional file 1 Experiment 1 data from Scaffold. Click here for file

Additional file 2 Supplementary tables. Click here for file

Additional file 3 Experiment 2 data from Scaffold. Click here for file

Additional file 4 Experiment 3 and 4 data from Scaffold, combined. Click here for file
