# Supplementary material for: The CJIE1 prophage of Campylobacter jejuni affects protein expression in growth media with and without bile salts
Source: BMC Microbiol. 2014 Mar 19;14:70. doi: 10.1186/1471-2180-14-70 (PMC4004267; doi:10.1186/1471-2180-14-70)
Supplement: Additional file 2 — Supplementary tables. [file 1471-2180-14-70-S2.docx]

**Supplementary Data File 2: Supplementary Tables**

Table S1. PCR primers used for assessing in isolate 00-2426 the presence and integrity of genes encoding proteins highly down-regulated in that isolate.

| **Primer set** | **Protein** | **Primer name** | **Primer sequence (5´-3´)** | Optimal annealing temperature (ºC) | Amplicon size (bp) |
| --- | --- | --- | --- | --- | --- |
|  | **Internal primers for gene presence/absence determination** | | | | |
| 1 | 2-amino-4-hydroxy-6-hydroxymethyl | DfolKf | AAA GGC AAA ATT ACA CTC ACT | 47.4 | 374 |
|  | dihydropteridine pyrophosphokinase | DfolKr | GCT CTT ATA GGC TTA GGA A |  |  |
| 2 | A/G-specific adenine glycosylase | AmutYf | GTG CGG TAC TTT CTA ACT CTT T | 48.7 | 511 |
|  |  | AmutYr | AAT ATA CGC CGT GTG CTT TCT C |  |  |
| 3 | ATP-dependent DNA helicase UvrD | Helf | YGA AAG CGA TGG YGG TGT AG | 50.8 | 460 |
|  |  | Helr | CTT GGC GCG AGT GAT GS |  |  |
| 4 | flagellar protein FliS | FliSf | TCA AGY GGG GAT AGA GTC | 46.8 | 245 |
|  |  | FliSr | TTG CCA AAG ATA AAA GTT |  |  |
| 5 | Holliday junction resolvase | C26f | GCT TTT CCT GTA ACG GCT TTT T | 50.8 | 201 |
|  |  | C26r | TTG CGA GGG TTT GGA CTT GAT |  |  |
| 6 | hypothetical protein Cj0566 | A566f | AAC GGT TTA CAT CTT TCA GGA | 47.4 | 441 |
|  |  | A566r | ATT GTC ATT GCG CTC ATA CTT A |  |  |
| 7 | hypothetical protein Cj0620 | A620f | TAT TTA TAA AGA TGA GTG CTA | 43.2 | 400 |
|  |  | A620r | AATGCTTAAATGCGTGTA |  |  |
| 8 | hypothetical protein Cj1429c | cj1429f | TTA AAT CCC CCT GCG ACA T | 49.3 | 491 |
|  |  | cj1429r | CAG AGG GGA TTT AAG GTG ATA GA |  |  |
| 9 | hypothetical protein CJE1531 | B1531f | ATT CCC ATG CCG TGA TT | 48.6 | 535 |
|  |  | B1531r | AAC ATG CTC CTT TTG ATA CTA |  |  |
| 10 | hypothetical protein CJJ81176_1118 | HypCJJf | ATA GAA TTT TCM ATA GTT G | 44.6 | 227 |
|  |  | HypCJJr | AYT TGA ATT TGC GTG ATA |  |  |
| 11 | hypothetical protein CJJ81176_1208 | C1208f | TTT TCT GCT TCA TTG GGT AT | 47.6 | 430 |
|  |  | C1208r | ATT CTT GGG AGC ACT TTG A |  |  |
| 12 | hypothetical protein ICDCCJ07001_659 | F659f | GCT ATA TTA ACG CTT TCT TT | 44.3 | 341 |
|  |  | F659r | ATT GCT TTA TTT ATT TAT TCT |  |  |
| 13 | hypothetical protein ICDCCJ07001_660 | F660f | CAG CAG CGG CAG CAA CA | 49.2 | 215 |
|  |  | F660r | AAA TAT TAC CGA GAC AAG GAA |  |  |
| 14 | hypothetical protein ICDCCJ07001_677 | F677f | CAG CCC CAC GAG GTA AAA T | 50.7 | 440 |
|  |  | F677r | TGG AAG CGC AAG TAA GAA A |  |  |
| 15 | hypothetical protein ICDCCJ07001_690 | F690f | AAA TTT GGG CGG TTG TAG AA | 50.9 | 392 |
|  |  | F690r | ATG GGC GAT TTT GTG ATA GG |  |  |
| 16 | invasion phenotype protein CipA | InvPf | CCT TTG ATT GGC GGA TTT CTT C | 50.9 | 431 |
|  |  | InvPr | TTG TTT TGC CTC CTG GGT TTT AC |  |  |
| 17 | methyltransferase family protein | Metf | TTT AAC TCC CCT ATC TTT TTC TCT | 48.3 | 489 |
|  |  | Metr | TCG TCC AGT ATG TAT TGA TTG TG |  |  |
|  | **For sequencing of upstream promoter regions** | | | | |
| 18 | Hypothetical protein Cj1429c | 81Cj1429plF | AAA AGA TTC GCC AAC CTA | 47.9 | 607 |
|  |  | 81Cj1429plR | AAG AGC TTT CGC ATC AAT |  |  |
| 19 | Flagellar protein FliS | FliSplF | AGG GTT TAR GTT GAA TTT TAG TGG | 50.8 | 619 |
|  |  | FliSplR | CTT CTC CGC CTT TTT CAT ART Y |  |  |
| 20 | ATP-dependent DNA helicase UvrD | HelplF | AAT TTC ACG CAG GTT TTG | 48.4 | 1154 |
|  |  | HelplR | TAT TTT CCC CAG TAT CTT TAG TG |  |  |
| 21 | hypothetical protein CJJ81176_1118 | HypCJJplF | RCC TTT AAC CAC CGC AGA A | 50.4 | 1037 |
|  |  | HypCJJplR | GCT TAC CGC TTG TTT TTG ACT ATC |  |  |
| 22 | invasion phenotype protein CipA | InvPplF | GCC ATC RAT AAA TTC AGC ACA A | 50.9 | 2252 |
|  |  | InvPplR | GCA GGG CAG GGC GTA AAG |  |  |
| 23 | methyltransferase family protein | MetplF | TTT TTA TAT CCA TTC AGC CAA CAT | 50.5 | 1413 |
|  |  | MetplR | TTC CGC CCA AGC CAC TAC |  |  |

Table S2. Effect of bile salts on the expression (log2 change versus MH agar as reference) of prophage proteins encoded by homologs of strain RM1221 prophages and integrated plasmid

| **Protein** | **GI Number** | MH + SD  fold change | | MH + OX  fold change | |
| --- | --- | --- | --- | --- | --- |
|  |  | 00-2425 | 00-2426 | 00-2425 | 00-2426 |
| **CJIE1 prophage** |  |  |  |  |  |
| ORF11 expressed by novel cargo gene in CJIE1 | gi\|313116388 | 1.33 ± 0.30 | ND | 1.10 ± 0.61 | ND |
| phage repressor protein, putative CJE0215 | gi\|157414969 | 0.30 (n = 1) | ND | -0.20 (n=1) | ND |
| phage major tail tube protein, putative CJE0226 | gi\|315124254 | 0.23 ± 0.60 | ND | 0.80 ± 0.62 | ND |
| hypothetical protein CJE0246 | gi\|315124241 | 0.63 ± 0.40 | ND | 1.13 ± 0.38 | ND |
| hypothetical protein ICDCCJ07001_690 CJE0228 | gi\|315124252 | -1.40 (n = 1) | ND | -1.00 (n = 1) | ND |
| extracellular deoxyribonuclease CJE0256 | gi\|57237266 | -1.13 ± 0.30 | ND | -1.47 ± 0.15 | ND |
| bacteriophage DNA transposition protein B CJE0269 | gi\|57237279 | 0.63 ± 0.30 | ND | 1.07 ± 0.75 | ND |
| hypothetical protein ICDCCJ07001_659 ORF7 | gi\|315124224 | 0.13 ± 0.3 | ND | 0.80 ± 0.10 | ND |
| signal peptidase I, putative CJIE1 prophage repressor associated with PanB | gi\|315124225 | 1.23 ± 0.20 | ND | 1.03 ± 0.29 | ND |
| **CJIE4 prophage** |  |  |  |  |  |
| phage integrase family site specific recombinase CJE1418 | gi\|57238154 | 1.07 ± 0.40 | 1.47 ± 0.12 | 1.23 ± 0.15 | 1.63 ± 0.29 |
| hypothetical protein CJE1429, putative phage repressor | gi\|57238165 | -1.47 ± 0.30 | -2.07 ± 0.61 | -2.07 ± 0.32 | -2.33 ± 0.50 |
| RloG protein, putative CJE1430 | gi\|57238166 | -0.67 ± 0.20 | -0.43 ± 0.23 | -0.07 ± 0.25 | 0.17 ± 0.32 |
| hypothetical protein CJE1439 | gi\|57238175 | 0.95 ± 0.5 | 1.15 ± 0.21 | 1.30 ± 0.42 | 1.45 ± 0.07 |
| signal peptidase I, putative phage repressor CJE1440 | gi\|57238176 | -0.23 ± 0.20 | -0.27 ± 0.31 | -0.50 ± 0.17 | -0.67 ± 0.21 |
| DNA/RNA non-specific endonuclease; CJE1441 | gi\|57238723 | -1.97 ± 0.90 | -1.83 ± 1.27 | -2.87 ± 0.71 | -2.63 ± 0.84 |
| hypothetical protein CJE1452 | gi\|57238311 | 0.2 (n=1) | 1.00 ± 0.30 | 2.00 (n=1) | 1.67 ± 0.87 |
| HK97 family major capsid protein CJE1458 | gi\|57238317 | 0.33 ± 0.40 | 0.87 ± 0.35 | 0.83 ± 0.90 | 1.80 ± 0.80 |
| hypothetical protein CJE1466 | gi\|57238325 | 0.83 ± 0.30 | 1.30 ± 0 | 1.47 ± 0.59 | 1.93 ± 0.74 |
| **CJIE2 prophage** |  |  |  |  |  |
| hypothetical protein CJE0598 (*C. jejuni* RM1221) | gi\|57238304 | 0.50 ± 0.40 | 0.77 ± 0.58 | 1.30 ± 0.14 | 1.17 ± 1.27 |
| **CJIE3 integrated plasmid** |  |  |  |  |  |
| cytochrome c family protein CJE1164 | gi\|153951605 | -2.07 ±0.40 | -2.53 ± 0.51 | -2.57 ± 0.21 | -2.93 ± 0.60 |
| high affinity branched-chain amino acid ABC transporter, ATP-binding protein CJE1158 | gi\|121613528 | -0.13 ± 0.10 | 0.07 ± 0.06 | 0.47 ± 0.23 | 0.47 ± 0.06 |
| high affinity branched-chain amino acid ABC transporter, periplasmic a.a-binding protein CJE1163 | gi\|121612759 | -2.47 ± 0.60 | -2.93 ± 0.35 | -2.87 ± 0.25 | -2.83 ± 0.15 |
| high affinity branched-chain amino acid ABC transporter, permease protein CJE1163 | gi\|157415273 | 0.97 ± 0.20 | 0.80 ± 0.10 | 0.72 ± 0.72 | 0.63 ± 0.38 |
|  |  |  |  |  |  |

Table S3. Effect of bile salts on the expression of selected known virulence proteins compared with growth on MH as the reference condition

| **Protein** | **COG** | **GI Number** | **Locus in NCTC 11168** | **0.1% sodium deoxycholate** | | **2.5% Oxgall** | |
| --- | --- | --- | --- | --- | --- | --- | --- |
|  |  |  |  | 00-2425 | 00-2426 | 00-2425 | 00-2426 |
| sialic acid synthase | M | gi\|218562755 | Cj1141 | -1.20 ± 0.10 | -1.50 ± 0.36 | -0.77 ± 0.75 | -1.00 ± 0.85 |
| capsular polysaccharide modification protein | M | gi\|218563017 | Cj1413c | 1.10 ± 0.20 | 1.33 ± 0.12 | 1.37 ± 0.25 | 1.47 ± 0.15 |
| capsular polysaccharide heptosyltransferase | M | gi\|218563035 | Cj1431c | 1.40 ± 0.30 | 1.60 ± 0.35 | 1.60 ± 0.26 | 1.63 ± 0.15 |
| capsular polysaccharide modification protein | M | gi\|218563018 | Cj1414c | 1.23 ± 0.20 | 1.27 ± 0.12 | 0.97 ± 0.35 | 0.93 ± 0.35 |
| capsular polysaccharide ABC transporter, periplasmic polysaccharide binding protein | M | gi\|157415670 | Cj1444c | 1.00 ± 0.40 | 1.37 ± 0.21 | 0.90 ± 0.36 | 1.13 ± 0.12 |
| capsular polysaccharide transport protein | M | gi\|157415671 | Cj1445c | 0.80 ± 0.20 | 0.90 ± 0.17 | 0.30 ± 0.44 | 0.37 ± 0.49 |
| capsular polysaccharide ABC transporter, ATP-binding protein | M, G, R | gi\|157415672 | Cj1447c | 1.17 ± 0.20 | 1.33 ± 0.15 | 1.43 ± 0.06 | 1.30 ± 0.17 |
| flippase in N-linked glycosylation | V | gi\|218562744 | Cj1130c | 1.10 ± 0.20 | 1.43± 0.06 | 1.17 ± 0.40 | 1.47 ± 0.25 |
| DnaK suppressor protein | T | gi\|153952045 | Cj0125c | -2.2 ± 0.40 | -3.10 ± 1.08 | -2.57 ± 0.55 | -2.93 ± 0.91 |
| sigma-54 associated transcriptional regulator | T | gi\|218562640 | Cj1024c | 0.80 ± 0.40 | 0.93 ± 0.15 | 1.03 ± 0.40 | 0.80 ± 0.36 |
| CheY chemotaxis protein | T | gi\|157415379 | Cj1118c | -1.97 ± 0.6 | -2.33 ± 0.23 | -2.10 ± 0.26 | -2.20 ± 0.17 |
| Peb1A bifunctional adhesin/ABCtransporter aspartate/glutamate-binding protein | T, E | gi\|157415178 | Cj0921c | -2.33 ± 1.00 | -3.33 ± 0.15 | -3.20 ± 0.35 | -3.40 ± 0.56 |
| CiaB |  | gi\|218562533 | Cj0914c | -2.03 ± 0.70 | -2.90 ± 0.17 | -2.23 ± 0.25 | -2.30 ± 0.17 |
| cytolethal distending toxin, subunit A | M | gi\|121613564 | Cj0079c | -0.47 ± 0.20 | -0.50 ± 0.17 | -0.77 ± 0.71 | -0.57 ± 0.59 |
| cytolethal distending toxin, subunit B | R | gi\|121612768 | Cj0078c | -0.90 ± 0.20 | -0.93 ± 0.96 | -0.63 ± 0.61 | -0.20 ± 0.26 |
| cytolethal distending toxin, subunit C |  | gi\|121613490 | Cj0077c | -0.85 ± 0.50 | -0.77 ± 0.38 | -0.10 ± 0.71 | -0.23 ± 0.45 |
| flagellar basal rod modification protein | N | gi\|121613172 | Cj0042 | -1.77 ± 0.80 | -2.87 ± 0.42 | -1.77 ± 0.38 | -2.33 ± 0.59 |
| FliA flagellar biosynthesis sigma factor | K | gi\|153952569 | Cj0061c | -1.23 ± 0.30 | -1.53 ± 0.70 | -1.50 ± 0.50 | -1.77 ± 0.76 |
| FlhF flagellar biosynthesis regulator | N | gi\|157414378 | Cj0064c | 0.77 ± 0.20 | 1.02 ± 0.15 | 1.07 ± 0.23 | 0.93 ± 0.40 |
| flagellar basal-body rod protein | N | gi\|157414984 | Cj0664 | 1.00 (n=1) | 0.50 ± 0.42 | 0.70 (n=1) | 1.00 ± 0.14 |
| FlaC | N | gi\|157415007 | Cj0720c | -1.10 ± 0.60 | -1.80 ± 0.57 | -1.40 ± 0.35 | -1.50 ± 0.14 |
| FlhA flagellar biosynthesis protein | N, U | gi\|121613700 | Cj0882c | 1.00 ± 0.10 | 1.13 ± 0.06 | 1.10 ± 0.07 | 1.20 ± 0 |
| flagellin modification protein PseA | D | gi\|121613681 | Cj1316c | -1.53 ± 0.70 | -2.03 ± 0.64 | -0.47 ± 1.12 | -0.63 ± 1.07 |
| flagellin modification protein A | I, Q, R | gi\|153952269 | Cj1332 | -1.03 ± 0.40 | -1.17 ± 0.21 | -0.93 ± 0.06 | -1.03 ± 0.23 |
| flagellin | N | gi\|57238729  gi\|218562948 | CJE1265  Cj1339c | 1.77 ± 0.02  4.0 | 2.17 ± 0.21  4.0 | 1.87 ± 0.15  4.3 | 2.07 ± 0.25  4.0 |
| flagellin | N | gi\|218562948 | Cj1339c | 1.82 ± 0.20 | 1.97 ± 0.12 | 2.00 ± 0.26 | 1.97 ± 0.12 |
| DnaJ | O | gi\|218562872 | Cj1260 | 0.63 ± 0.10 | 0.83 ± 0.06 | 0.80 ± 0.36 | 0.67 ± 0.57 |
| GroEL chaperonin | O | gi\|121612249 | Cj1221 | -0.90 ± 0.50 | -1.13 ± 0.74 | 0.47 ± 0.21 | 0.87 ± 0.31 |
| GroES co-chaperonin | O | gi\|157415483 | Cj1220 | -1.73 ± 0.50 | -2.07 ± 0.21 | -2.87 ± 0.15 | -2.93 ± 0.25 |
| TlyA putative hemolysin | J | gi\|218562239 | Cj0588 | 1.10 ± 0.50 | 1.33 ± 0.38 | 1.03 ± 0.46 | 1.23 ± 0.35 |

COGs: [D] Cell cycle control, cell division, chromosome partitioning; [E] Amino acid transport and metabolism; [G] Carbohydrate transport and metabolism; [I] Lipid transport and metabolism; [J] Translation, ribosomal structure, and biogenesis; [K] Transcription; [M] Cell wall/membrane/envelope biogenesis; [N] Cell motility; [O] Posttranslational modification, protein turnover, chaperones; [Q] Secondary metabolites biosynthesis, transport and catabolism; [R] General function prediction only; [T] Signal transduction mechanisms; [U] Intracellular trafficking, secretion, and vesicular transport; [V] Defense mechanisms
